# Nanomedicine and versatile therapies for cancer treatment

**DOI:** 10.1002/mco2.163

**Published:** 2022-08-18

**Authors:** Aparna Shukla, Pralay Maiti

**Affiliations:** ^1^ School of Materials Science and Technology Indian Institute of Technology (Banaras Hindu University) Varanasi India

**Keywords:** administration routes, cancer, controlled drug delivery, nanomedicines

## Abstract

The higher prevalence of cancer is related to high rates of mortality and morbidity worldwide. By virtue of the properties of matter at the nanoscale, nanomedicine is proven to be a powerful tool to develop innovative drug carriers with greater efficacies and fewer side effects than conventional therapies. In this review, different nanocarriers for controlled drug release and their routes of administration have been discussed in detail, especially for cancer treatment. Special emphasis has been given on the design of drug delivery vehicles for sustained release and specific application methods for targeted delivery to the affected areas. Different polymeric vehicles designed for the delivery of chemotherapeutics have been discussed, including graft copolymers, liposomes, hydrogels, dendrimers, micelles, and nanoparticles. Furthermore, the effect of dimensional properties on chemotherapy is vividly described. Another integral section of the review focuses on the modes of administration of nanomedicines and emerging therapies, such as photothermal, photodynamic, immunotherapy, chemodynamic, and gas therapy, for cancer treatment. The properties, therapeutic value, advantages, and limitations of these nanomedicines are highlighted, with a focus on their increased performance versus conventional molecular anticancer therapies.

## Graphical abstract

Design of nanocarriers and drug delivery vehicles along with advanced therapeutics for cancer treatment.

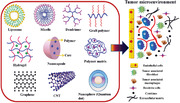

## 1 INTRODUCTION

Developing effective treatments and devices for disease control has been a strong desire over the years. Immense understanding about the functioning of the human body and its components (organs, bones, muscle blood, etc.) are underway for prevention and cure. Knowledge of living cells and physiology in general has brought about advancements in several medicines using both natural and synthetic materials to combat infections, aliments, and malfunctions of the human body. Cancer is considered one of the malignant diseases and is the foremost reason for higher fatality rates in most countries. The term “cancer” represents the unlimited growth of cells and their multiplication. These cells have enormous replication potential, prompt angiogenesis, and promote invasion and metastasis, which tagged this as “most dreaded disease” in the world. There are various types of cancers with few representative or familiar characteristics, making its treatment demanding.^1^ Cancer is such a deadly disease that can affect any of the body parts, but the lung, female breast, prostate, and liver are more prone to infection. Unique characteristic features of cancer are its fast and unlimited growth rate of cells in an uncontrollable manner in body organs, leading to malignant tumors, which are the major cause of mortality. Different factors are responsible for causing cancer; few of them are physical mutagens, such as ultraviolet and ionizing radiation; chemical mutagens, such as asbestos, tobacco, and arsenic; and biological mutagens, which include infections from viruses, bacteria, and parasites.

According to the World Health Organization, approximately 30–50% of cancer deaths can be avoided by adopting three different strategies: consciousness, clinical diagnostic techniques, and care.^2^ For the past 10 years, substantial efforts have been made in cancer therapy. Traditional treatment of cancer embraces surgery, chemotherapy,^3^ and radiation therapy, but these techniques bear some limitations.^4^, ^5^ The most common treatment of cancer is conventional chemotherapy, while its efficacy is reduced due to its nonspecificity and quick elimination of many anticancer drugs, lower efficiency, drug resistance, and the toxicity induced by the chemotherapeutics when administered frequently and at higher doses.

Nonetheless, the detrimental effect of chemotherapeutics is damage to normal cells, which affects the immune system and leads to side effects, such as loss of craving, alopecia, and sickness. The prime cause of such intense unfavorable fallout and higher mortality rates is the excessive dose of chemotherapeutics beyond their remedial limit in normal healthy tissues and delicate body parts after administration originating due to burst release of drugs.^6^ Another important factor is the poor bioavailability of most anticancer drugs due to their electronegative surfaces or zeta potential, which means the drugs are forced back by the negative charge at the cytomembranes, resulting in inadequate adhesion of cells and ultimately insignificant bioavailability.^7^ This motivates medical practitioners to administer a higher dose of drug than the required dose to maintain diffusion‐controlled phenomena. Therefore, targeted drug delivery carriers for cancer treatments are currently more fascinating, as they can improve remedial and diagnostic efficiency and thereby minimize adverse side effects.^8^ This prompted researchers to develop chemotherapeutics that can passively or actively target cancer cells, thereby minimizing detrimental side effects and enhancing therapeutic efficiency. There is a need for designing and developing controlled drug delivery systems that can release the drug in a controlled manner for an extended period to maintain the therapeutic concentration.^9^

In this review, the focus is on different drug delivery vehicles (organic and inorganic) for cancer treatment, their advantages over traditional methods of treatment, and why there is a need for control drug delivery systems. The different modes of administration (different therapies used) of these nanocarriers loaded with cargo and their potential as immunotherapeutic targets in the future have also been highlighted.

### 1.1 Adverse effects in conventional therapies

For better absorption, the solubility of chemotherapeutics plays a significant role, as they must be soluble in blood either administered intravenously or given orally. Hydrophobicity and poor solubility of the chemotherapeutics in aqueous medium worsen their therapeutic efficiency. Furthermore, most anticancer drugs are identified as foreign particles by macrophages and can be digested or engulfed by them, resulting in poor therapeutic effects. Because of the nonselective nature of anticancer drugs, normal healthy cells are also affected, which is the prime reason for higher death rates in cancer patients. Adverse effects included blood‐related side effects, loss of appetite, hair loss (alopecia), nausea, and vomiting. To circumvent these obstacles, targeted chemotherapy has emerged as a novel approach to reduce the limitations and nonspecificity of conventional chemotherapies. In an ideal drug release system, the drug is delivered in vivo at its therapeutic dose and selectively kills cancerous cells. For many instances, such drug release is not facile, as there are some delivery barriers, such as degradation of the drug by enzymes, activation of the immune system, morphological barriers, approach to tissues or cells, nephritic and liver clearance, fast release, and induced toxicity. To avoid such hurdles, control drug release carriers are the need of the hour and are being designed.

Biomaterials designed for cancer treatment have been extensively developed and are still being explored in different areas. Among such systems, photosensitive materials play an important role. Photosensitive materials are divided into two primary classes: photothermal and photodynamic materials. In general, light or photons are converted into heat and thermal energy, which kill tumor cells in photothermal therapy (PTT). The heat generated due to energy transition increases the local temperature high, resulting in killing of tumor cells without affecting the normal cells. Owing to the unique characteristics of photothermal conversion, these functional materials are preferred in biomedical applications.^10^, ^11^, ^12^ Photodynamic therapy (PDT) is another mode of cancer treatment that uses photosensitizers and light activation. Irradiation with light of appropriate wavelength activates the photosensitizing (PS) drugs selectively present in tumor tissues, which generate a cascading photochemical reaction that in turn damages the tumor cells. PS drugs produce highly active singlet oxygen species that cause toxicity and ultimately kill tumor cells.^13^, ^14^ PDT offers effective treatment with minimal side effects and is known to cause immunogenic cell death in cancer cells.^15^, ^16^ PTT has gained importance with its rapid growth in cancer treatment but has yet to be applied clinically as the life span and diffusion distance of reactive oxygen species (ROS) are less, and low oxygen and poor penetration cause damage to normal tissues.^14^, ^17^, ^18^

### 1.2 Requirement of sustained release for disease control

**FIGURE 1:**
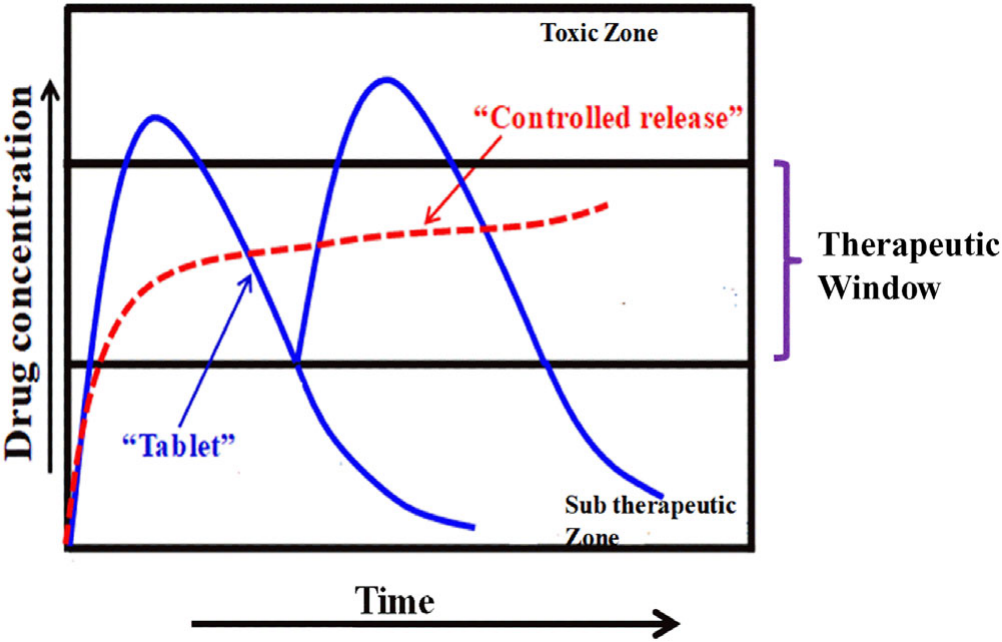
A schematic presentation of controlled release systems, variation of drug concentration in blood stream as a function of time, comparing the traditional release system

Control drug delivery has become one of the steadily flourishing fields of medical sciences combining people from all around different specializations, such as chemistry, materials science, chemical engineering, biology, and medicine, particularly those working in the area of the health care sector.^19^ The control drug release system provides superior efficiency, lowers toxicity, and improves patient compliance. The prime objective of control release vehicles is enhancing the efficacy of drug release, which in turn results in improving the therapeutic efficacy by eliminating the adverse toxicity due to the drug and the dosage of drug consumption during treatment.^20^ The advent of controlled drug release systems over the past few years has been the most fascinating and has gained significant momentum, especially in the pharmaceutical and health care sectors. It has now become one of the significant multidisciplinary studies due to strenuous efforts. As a consequence of the advantages of control drug release systems, including efficiency, safety, cost economy, and better patient compliance, over traditional treatment methods has led to a significant increase in the works in this field. Normally, the control drug delivery systems are defined as the targeted entities to deliver the drug/chemotherapeutics at a particular site at a pre‐established rate for a longer duration of time. A control release system (CRS)^21^ is challenging since there is a need for a physical substance in which the desired curative of a specific amount could be placed safely, preventing the therapeutic from its early breakdown before release, and is likely to release the therapeutics over a duration of time (Figure 1).^22^ Materials required for CRS must be biomaterials, and they should possess the important criteria of biocompatibility, easy processability, and sufficient mechanical strength.^23^

To date, various techniques for the delivery of therapeutics at the desired site have been reported, which not only improve the efficacy of chemotherapeutics but also minimize the related side effects. The delivery vehicles used generally consist of matrices and reservoirs, such as biodegradable, bioresorbable materials and hydrogels. Current progress in polymer chemistry and the evolution of new polymerization approaches has enabled the generation of polymers with well‐designed structures with narrow molecular weights and tunable properties.^24^, ^25^, ^26^ Similarly, recent advances in nanotechnology have resulted in the production of nanoparticulate carriers with narrow distribution and administrable physiochemical properties, which can further be exploited for different causes, such as monitoring the efficacy of treatment and improving its efficiency. Important reasons behind the application of polymers and nanoparticles as drug delivery vehicles are their ability to increase the aqueous solubility of drugs and enhance their circulation period in the blood, thereby eliminating their renal excretion.^27^

## 2 NANOCARRIERS FOR CONTROLLED DRUG RELEASE FOR CANCER TREATMENT

**FIGURE 2:**
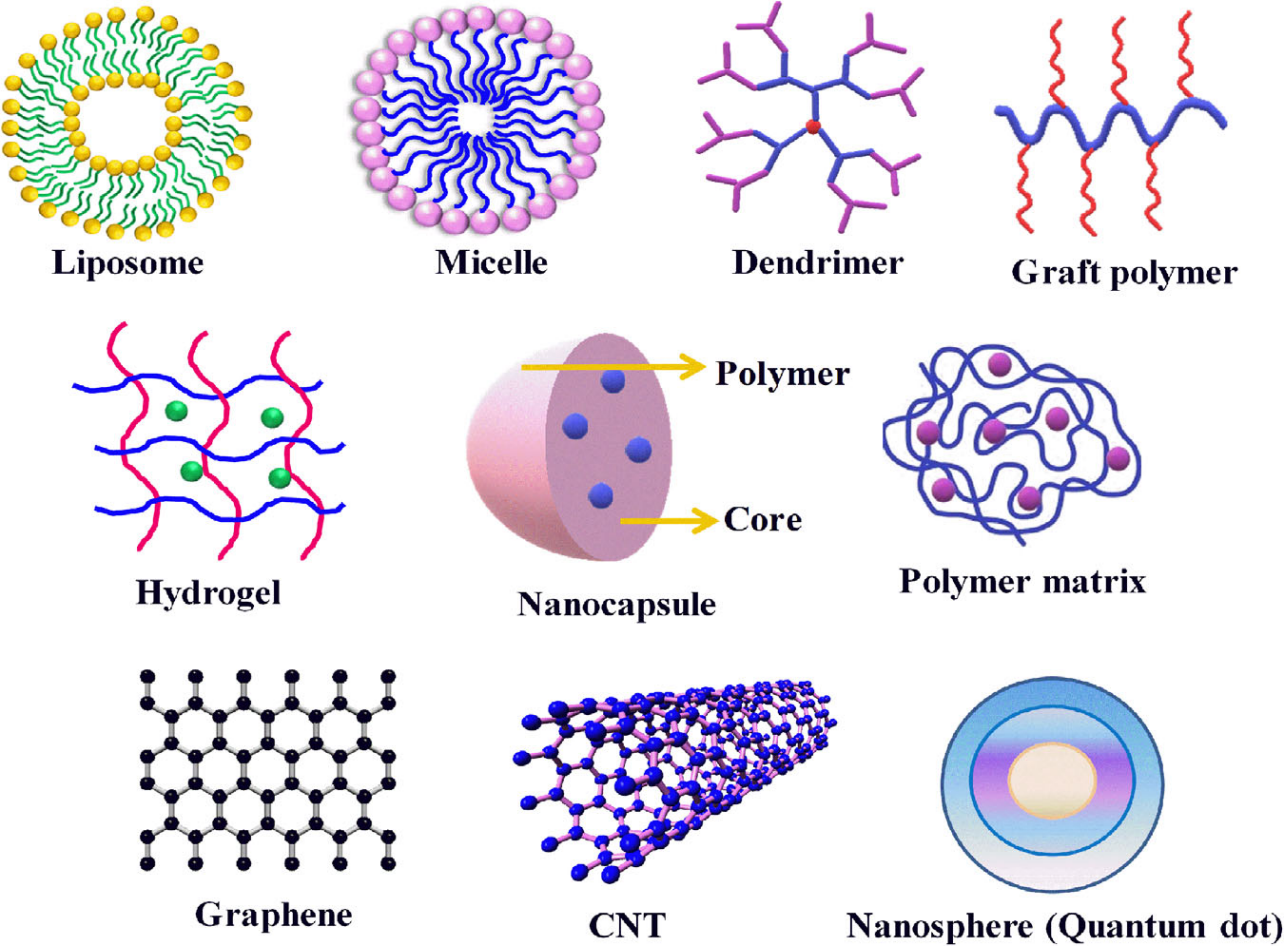
Different nanocarriers, with their shape and size, used in control drug delivery systems

In controlled drug delivery settings, polymers have emerged as the most fascinating materials with a long‐standing role as drug carriers for the cure of cancer. Numerous polymer‐based drug carriers^28^ have been explored in the literature thus far, including organic and inorganic nanocarriers, where organic nanocarriers consist of polymer drug conjugates, dendrimers, liposomes, polymeric micelles, electrospun scaffolds, micro/nanogels, block or graft copolymer‐based nanoassemblies, while inorganic nanocarriers include carbon‐based systems (carbon nanotubes [CNTs] and graphene oxide [GO]) and magnetic nanoparticles (iron oxides), as presented in Figure 2.^29^ Furthermore, the infusion of the abovementioned drug carriers inside the cytoplasm of the cell is one of the crucial concerns for the better potency of drugs against tumor treatment. Usually, there is a large gap between epithelial cells in the blood vessels in cancerous tissues, resulting in defective vascular architecture and inferior lymphatic drainage. Nanocarriers can extravasate across these gaps and can be assembled in tumor tissue, and this process is termed the enhanced permeability and retention (EPR) effect. Broader applications of polymers stem from the fact that they possess readily tunable properties from a chemical point of view; for example, the molecular weight and structure of polymers can easily be controlled by different strategies, such as ATRP,^30^ RAFT,^31^ NMO, and ROMP. Polymers^32^ belong to a versatile category of materials that are omnipresent in the modern world.^33^

### 2.1 Advantages of nanocarriers in cancer delivery

Drug delivery through nanoparticulate‐based systems offers different advantages for cancer treatment against pure drug administration. They work by enhancing the therapeutic index of the embedded chemotherapeutic agents in these nanocarriers compared to the conventional delivery of drugs, by improving the efficacy of the drug in attaining steady‐state therapeutic levels for a prolonged time period, and by reducing the drug toxicity due to sustained/controlled release of the drug, further improving drug pharmacokinetics by increasing its stability and solubility.^34^ There are other certain advantages from the engineered nanocarriers compared to free drug administration, such as their nanometer size dimension appropriate for tumor targeting via the EPR effect, protective shielding of the drug, thereby enhancing its stability and minimizing its fast clearance, ease of surface modification, and feasibility of multiple drug delivery to achieve synergistic effects, and most importantly provide a scope of combination therapy by exploiting chemotherapeutic and photothermal effects or creating magnetic nanostructures.^35^ Additionally, nanocarriers loaded with chemotherapeutic agents reduce chemoresistance to drug action by selectively targeting cancer cells and imparting no toxicity to normal cells.^36^ Different organic/inorganic carriers are discussed below.

#### 2.1.1 Graft polymers

**FIGURE 3:**
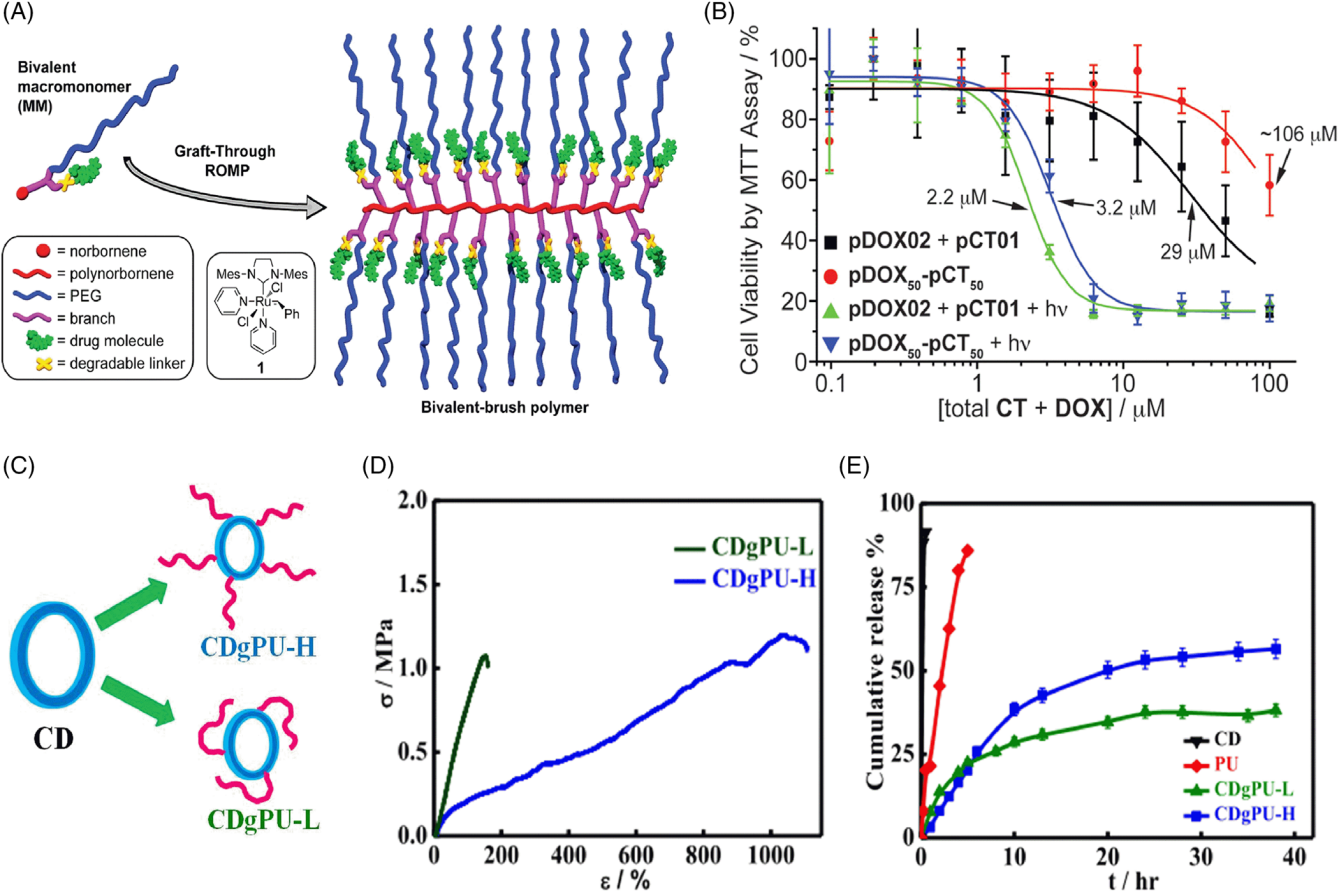
Graft polymers for chemotherapeutic delivery. (A) Schematics of bivalent macromonomer (MM) and bivalent‐brush copolymer; (B) cell viability of MCF‐7 human breast cancer cells treated with drug‐loaded brush polymers both with and without UV irradiation, showing the IC_50_. Reproduced with permission from Ref. ^49^ Copyright 2010 American Chemical Society; (C) grafting of polyurethanes onto CD yielding different graft density (low and high) copolymers; (D) mechanical properties of prepared graft copolymers; and (E) drug release profile for pure CD and its developed graft copolymers as indicated. Reproduced with permission from Ref. ^51^ Copyright 2019 Elsevier

To date, a large number of polymers with different topologies, such as block, gradient, star, hyperbranched, dendritic, cyclic, and graft, have been chemically synthesized successfully.^37^ Studies and applications of such polymers allow one to explore new functionalities and properties of these materials and the ability to tune the chemical properties through designs.^38^ In general, graft polymers^39^ possess multiple side chains on linear polymeric backbones that are attached chemically and are equipped with captivating properties having worm‐like compact molecular structures. Increasing attention and importance are given to the development of these macromolecular systems in understanding their architectures, properties, and potential applications.^40^ Graft copolymers^41^ with well‐defined architecture having desired functionalities, chemical compositions, graft length, and graft densities have widely been used in biology and nanoscience. To date, three different strategies have been employed for the synthesis of graft copolymers and are termed “grafting through,”^42^ “grafting,” and “grafting onto.”^43^ Drug delivery using densely grafted molecular designs, especially brush polymers, has gained considerable importance in recent years. Diblock grafts of amphiphilic PCL‐b‐PEG poly(ε‐caprolactone)‐b‐poly(ethylene glycol) brush polymers have been explored for the confinement and release of the anticancer drug doxorubicin (DOX).^44^, ^45^ Brush copolymers with block structures have been developed for drug delivery; for example, PEG and cholesterol containing amphiphilic diblock brush copolymers have been reported to show higher weight percentage encapsulation of DOX in these copolymers with enhanced delivery of drug at the tumor site.^46^, ^47^ Brush polymer–drug conjugates have also been prepared and studied by Johnson et al. with repeating backbones having PEG chains and drug moieties (DOX and paclitaxel [PTX]), which can be used in chemotherapy.^48^, ^49^ A schematic presentation of bivalent macromonomer and bivalent‐brush polymers is presented in Figure 3A, where a PEG side chain used is water soluble and drug moieties are connected via a branch point to a polynorbornene backbone. The success of grafting through ROMP has been proven through gel permeation chromatography and nuclear magnetic resonance (NMR) studies. The anticancer drugs camptothecin (CT) and DOX are conjugated using a degradable linker that facilitates sustained drug release in the presence of a stimulus. The developed system combined with the versatility of graft‐through ROMP is a novel approach to incorporate new cleavable linkers into such polymers.

Upon irradiation, drug‐conjugated polymers exhibit significant toxicity against MCF‐7 human breast cancer cells (IC_50 _= 2.2 and 8.7 μM for CT‐bound polymer and DOX‐bound polymer, respectively) compared with the nonirradiated samples illustrating photoinitiated release (Figure 3B). The brush copolymers carrying both CT and DOX display a 30‐fold increase in toxicity upon irradiation. Grafted polyurethanes onto a cyclodextrin (CD) backbone with varying graft densities (low as well as high graft density) are presented in Figure 3C together with varying graft length^50^ to control the release of an anticancer drug (dexamethasone) in a sustained manner. The grafting is confirmed through the NMR technique and is well matched with molecular weight estimation through gel permeation chromatography. The prepared grafted systems are thermally and mechanically stable (Figure 3D) and possess enough strength for their application in biomedical applications. An in vitro sustained drug release pattern is exhibited from these copolymers against burst release from pure drug, as presented in Figure 3E. From cellular studies, cell viability gradually decreases with time, and approximately 80% cell mortality is observed after 5 days using graft copolymers, while a meagre killing is observed using pure drug due to its burst release pattern, which is very well reflected in the in vivo melanoma model, where a reduction in tumor volume is observed after treatment using the developed graft copolymeric patch. Moreover, the body weight of mice increased with time after treatment with the graft patch against a consistent decrease in the pure drug‐treated systems. Furthermore, no side effects on vital organs were observed in histopathological studies, indicating the efficacy of these CD‐grafted polyurethane systems for biomedical applications.^51^ Mahanta et al. prepared polyurethane‐grafted chitosan copolymers with various degrees of substitution for sustained drug delivery. These grafted systems are found to be better biocompatible materials and control drug release compared to native chitosan, showing a Fickian mode of diffusion (*n* ≤ 0.45). The rate of release is governed by the degree of polyurethane chains substituted onto chitosan. These graft copolymers are hemocompatible, as observed through platelet aggregation, cellular and hemolysis studies.^52^ Different polymeric materials reported for chemotherapeutic delivery are presented in Table S1.

#### 2.1.2 Liposomes

Usually, liposomes are constituted from either one or two lipid bilayers and have a spherical morphology. Liposomes^53^ are basically used in the delivery of both lipophilic and hydrophilic drugs, where the lipid bilayer incorporates the lipophilic drug and the inner aqueous core stabilizes the hydrophilic drug. The US Food and Drug Administration (FDA) in 1995 approved PEGylated liposomes with DOX, that is, doxil PEG incorporation on the liposomal surface enhances the half‐life circulation, thus taking advantage of the EPR effect.^54^ Instead of PEG, various hydrophilic polymers, such as poly(N‐vinyl pyrrolidone) (PVP), poly(vinylalcohol) (PVA), polyoxazoline (Pox), hyperbanched polyglycerol, or zwitterionic polymers, have also been employed.^55^, ^56^ Wu and coworkers prepared a transferrin‐conjugated liposome by entrapping DOX and varapamil, a P‐gp inhibitor. The efficacy in K562 cells has been evaluated, and the significant toxic effects caused by overcoming P‐gp‐mediated multidrug resistance have been demonstrated.^57^ Another report optimized smart nanoparticles in which liposomes were doubly loaded to attain improved tumor efficiency. The aqueous part is laden with iron oxide nanoparticles, and the lipid part is endowed with a chemically initiated photochemical reaction. These developed liposomes with dual functionality address both chemotherapeutics inside tumor cells and combined PDT/hyperthermia emanated in complete destruction of cancer cells in vitro, while abolition of solid tumors in an in vivo model.^58^ Ta et al. prepared polymer‐modified thermosensitive liposomes composed of temperature‐responsive N‐isopropylacrylamide (NIPAAm) and pH‐responsive polyacrylic acid for the delivery of DOX. These liposomes manifest in an enhanced release profile and significantly lower thermal dose threshold and are stable in serum with minimal drug leakage over time.^59^ Liu et al. fabricated 3D bioprinted patches composed of fish gelatin methacryloyl (F‐GelMA) and PEGylated liposomal dox incorporated into hydrogel as a nanomedicine. Carboxymethyl cellulose was added to increase the viscosity of F‐GelMA and inhibit the increase in particle size in F‐GelMA hydrogels. The release of drug from 3D‐printed patches was regulated through the shape of the patches and their UV influence time, which can be controlled easily.^60^

#### 2.1.3 Hydrogels

**FIGURE 4:**
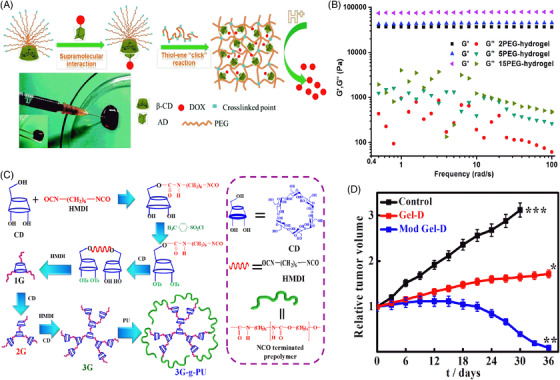
Different hydrogels for the delivery of chemotherapeutics. (A) Preparation of injectable DOX‐loaded hydrogels based on host–guest supramolecular interactions between CD and AD. (B) Rhelogy of different PEG hydrogels. Reproduced with permission from Ref. ^64^ Copyright 2017 Royal Society of Chemistry. (C) Schematics of different generations of CD using small spacer HMDI and grafting with polyurethane forming superstructure (3G‐PU), embedded in methyl cellulose making whole system as injectable gel; (D) in vivo melanoma studies after the treatment with prepared injectable gels. Reproduced with permission from Ref. ^65^ Copyright 2019 American Chemical Society

Hydrogels are three‐dimensional (3‐D) networks constituted by cross‐linked hydrophilic polymeric chains. Due to ease of fabrication, biocompatibility, tunable composition, and superior physical properties have made these materials promising for tremendous biomedical applications. The basic objective of hydrogel‐based technology is the development of injectable hydrogels,^61^, ^62^ where the gel precursor, usually aqueous, is blended with other biopolymers or biologically active agents and then administered via syringe at the desired area of interest. The prime advantage of injectable hydrogels rests on their highly flexible properties (acquiring the desired shape), when applicable in vivo results in fast recovery with smaller scar size and minimum pain caused to the patients, retaining higher capacity and enhanced drug or gene encapsulation for their delivery. For the targeted and localized delivery of drugs inside tumorous cells, in situ gelation of injectable hydrogels has been proven to be more effective, as it preserves the enclosed drugs inside the tumor and incisively liberates the drugs into tumorous cells.^63^, ^64^ Drug delivery through hydrogels can be attained in different ways, such as oral, rectal, ocular, epidermal, and subcutaneous (SC) administration. CD and PEG modified with gold nanocrystals forming supramolecular hydrogels demonstrate pH‐dependent release of drug arising from host–guest interaction of DOX with CD. These DOX‐loaded microgels displayed systematic antitumor effects toward HeLa cells compared to pure microgels. Supramolecular host–guest interactions between CD and adamantane (AD) are well explored for the development of supramolecular hydrogels that possess self‐healing, shape memory, and injectable properties. Based on this approach, Sheng et al. developed dendron‐like multifunctional β‐CD‐PEG conjugates with several PEG arms that are terminated with acrylates. DOX, a potent hydrophobic anticancer drug, has been modified with AD (AD‐DOX) through a benzoic imine bond and is added to the CD‐PEG hydrogel precursor simply by mixing, where host–guest interactions between CD and AD occur and are further crosslinked with poly[oligo(ethylene glycol) mercaptosuccinate] (POEGMS), resulting in injectable gels. A schematic is presented in Figure 4A. The product in each and every step was confirmed through ^1^H NMR. Rheological studies showed that G’ values are independent of frequencies, confirming the stability of the crosslinked hydrogel network (Figure 4B). Moreover, the storage modulus increases with increasing PEG content due to higher crosslinking. DOX release from the gel occurs after the cleavage of the benzoic‐imine bond between AD and DOX in an acidic environment in tumors, leading to cell killing.^64^ Furthermore, the development of an injectable gel, by preparing different generations of CD and subsequent grafting of polyurethanes, and finally embedding the copolymers in methyl cellulose solution converted the whole system into an injectable gel (Figure 4C). Gel shows enhanced drug (PTX) release with complete melanoma shrinkage after 30 days of treatment (Figure 4D). Histological analysis reveals that injectable gel is safe and no side effects are observed on vital organs.^65^ For minimally invasive delivery, smaller hydrogel particles can be used, which are termed nanogels or microgels. Owing to their smaller size (a few nanometers), they not only facilitate easy needle injection but also provide a greater surface area for conjugation with biomolecules and more penetration into biological tissues. Nanogels are prominently used for the delivery of DNA used in gene therapy, which is very promising for the treatment of cancers, hemophilia, and other viral diseases.^66^

Polymer‐protein conjugated nanogels are reported and are known for enhancing plasma half‐life and protein stability.^67^ Lee et al. prepared injectable biodegradable hydrogels through phase separation between PBA‐functionalized polycarbonate and PEG‐based triblock copolymer for controlled delivery of BTZ. pH‐dependent in vitro sustained release of BTZ is observed from the composite hydrogel. These composite hydrogels exhibit antitumor effects that are enhanced after administration of a single SC dose of BTZ‐loaded micelle/hydrogel composite against BTZ‐loaded micelle solution.^68^

#### 2.1.4 Dendrimer

Recently, polymers with high branching have emerged in pictures possessing properties quite different from the respective linear entity. Their unique properties arise from complicated dendritic/hyperbranched structures having multiple chains whose ends are highly branched, leading to new physical properties. Their architecture offers advantages and finds their application in drug release systems. Interior as well as peripheral regions on dendrimers can be utilized for host–guest reactions. Dendrimers have a hyperbranched 3D architecture possessing higher surface versatility and functionality. Since their emergence in the 1980s, dendrimers have been very promising polymeric materials owing to their unparallel properties, such as their uniform size, aqueous solubility, nanoscale size, low polydispersity, and well‐defined molecular weight distribution. The inner cavity is the place where particular guest molecules can be encapsulated primarily small drug molecules, while the outer peripheral part with different functionalities can actively conjugate with biological agents. These attractive features have made dendrimers much more fascinating for drug delivery applications.^69^ Predominantly used dendrimers in drug delivery systems are polyamidoamines PAMAM, poly(L‐lysine) PLL, polyesters PGLA‐OH, polypropylimines, and some citric acid‐carbohydrate‐based polymers. Tekade et al. prepared a polyamidoamine dendrimer and encapsulated the dual drugs methotrexate (MTX), a hydrophobic drug, and all‐trans retinoic acid, a hydrophilic drug, and showed enhanced cytotoxicity caused by dendrimers toward HeLa cells in comparison to the free drug, and hemolytic toxicity was also reduced.^70^ Luong and coworkers synthesized 3,4‐difluorobenzylidene diferuloylmethane (CDF)‐loaded folate‐conjugated PAMAM‐based carriers for the treatment of cervical and ovarian cancer. In a cell study, FA‐PAMAM‐CDF nanocarriers exhibited greater resistance toward cancer cells than pure CDF and FA‐PAMAM.^71^ Thomas et al. explored folic acid (FA)‐ and MTX‐conjugated PAMAM for tumor treatment, wherein polyvalent MTX plays a dual role in the nanocarrier as a targeting agent and as an anticancerous drug. From in vitro cell toxicity data, higher drug content loaded (10 wt.%) FA‐PAMAM nanocarriers displayed 65% killing after 2 days, while lower drug loaded (5 wt.%) FA‐PAMAM nanocarriers showed 45% killing in 2 days due to suppression of dihydrofolate reductase.^72^

#### 2.1.5 Micelles as drug carriers

**FIGURE 5:**
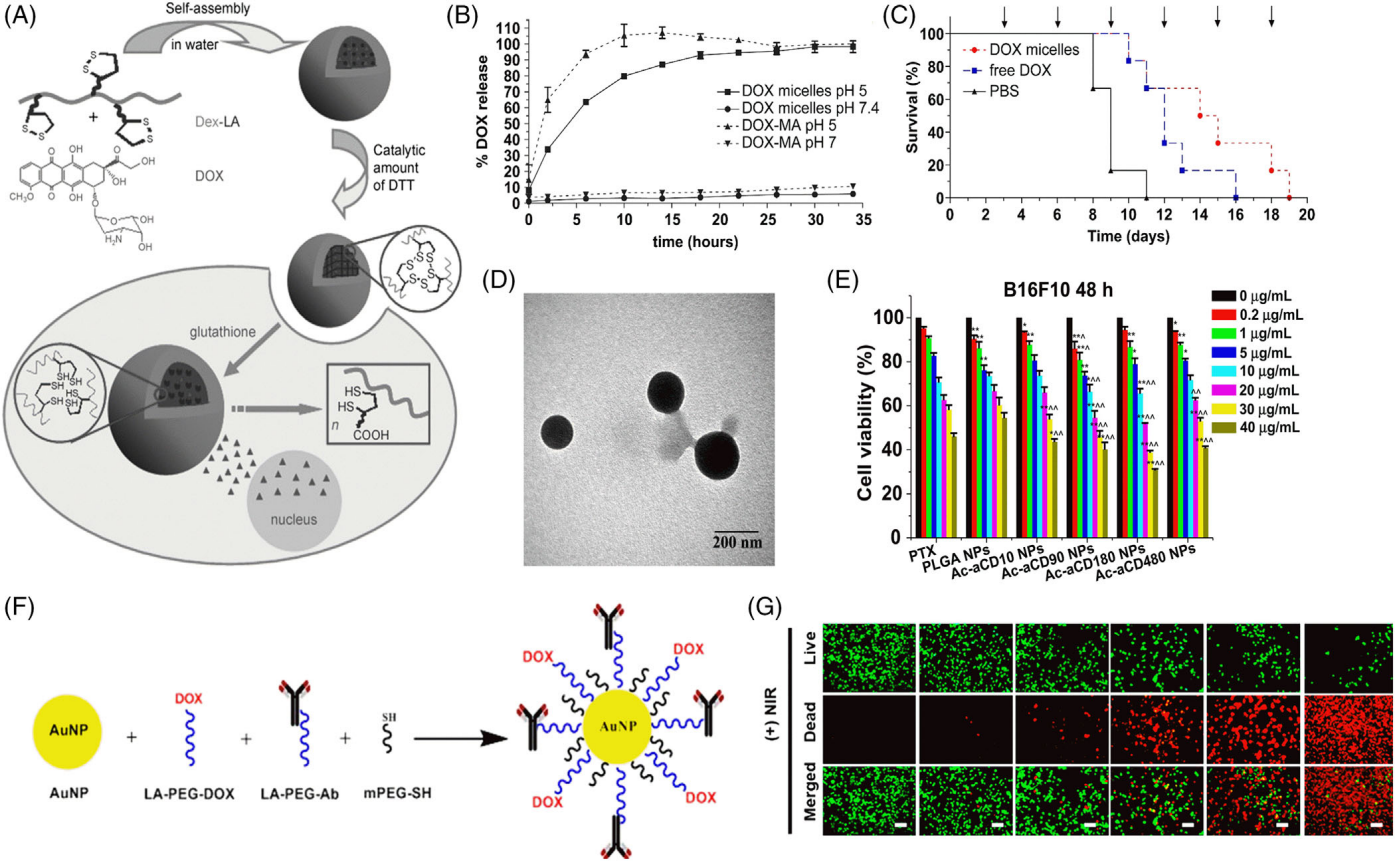
Nanoparticles for the delivery of anticancer drugs. (A) Scheme showing reversibly stabilized multifunctional dextran‐lipoic acid (Dex‐LA) nanoparticles. Reproduced with permission from Ref. ^78^ Copyright 2009 Wiley. (B) In vitro release of DOX from PEG‐b‐p(HPMAm‐Lacn) micelles at pH 5 and 7.4 at 37°C; (C) percent survival of mice‐bearing B16F10 melanoma carcinoma after administration of PBS, free doxorubicin (3 mg/kg), and micelles with covalently bound DOX (3 mg/kg). Reproduced with permission from Ref. ^79^ Copyright 2010 Elsevier. (D) TEM images of PTX/Ac‐CD nanoparticles. (E) In vitro cytotoxicity of PTX, PTX‐loaded PLGA NPs, and PTX‐loaded Ac‐aCD NPs against B16F10 cells after 48 h of incubation. Reproduced with permission from Ref. ^88^ Copyright 2013 Elsevier. (F) Schematic reaction involved in conjugation of LA‐PEG‐DOX, LA‐PEG‐PD‐L1, and PEG‐SH onto the surfaces of AuNP; (G) live/dead staining assay results of the effects of NT‐AuNP, PD‐L1‐AuNP, NT‐AuNP‐DOX, PD‐L1‐AuNP‐DOX, or DOX (0.5 μg/ml) treated for 24 h with or without NIR. Reproduced with permission from Ref. ^90^ Copyright 2019 American Chemical Society

Self‐assembly of polymers with hydrophilic and hydrophobic (amphiphilic) blocks in aqueous solution results in the formation of micelles with hydrophobic cores and hydrophilic shells forming globular or spherical shapes.^73^ The hydrophobic drug resides in the hydrophobic core, while the stability to the hydrophobic core and hydrophobic drug is provided by the hydrophilic shell, making the particles of appropriate size for intravenous (IV) administration. Drug incorporation into polymeric micelles is usually performed through physical, chemical, or electrostatic interactions.^74^ Delivering drugs simultaneously through micelles has been reported in the literature for effective tumor treatment. Polymer‐based micelles composed of amphiphilic block copolymers poly(2‐methyl‐2‐oxazoline‐b‐2‐butyl‐2‐oxazoline‐b‐2‐methyl‐2‐oxazoline)(P(MeOx‐b‐BuOx‐b‐MeOx) loaded simultaneously with two drugs PTX and alkylated cisplatin prodrug have been used for combination therapy of ovarian and breast cancer.^75^ In another work, they connected DOX by a hydrazone bond to an amphiphilic highly branched block copolymer composed of a hyperbranched polyester Boltron H40 core, constituting poly(aspartate) as an aquaphobic part and constituting PEG outside. The acidic environment facilitates the cleavage of hydrazone linkages between poly(L‐aspartate) and DOX and causes its release.^76^ In another report, Shin et al. developed a block copolymeric micelle (PEG‐b‐PLA) as a nanocarrier for three different poorly water‐soluble drugs (PTX, 17‐AAg, and rapamycin). This micelle nanocarrier of three drugs showed a cooperative effect in MCF‐7 and 4T1 breast cancer cells, an effective formulation for cancer therapy.^77^ To date, strong micelles have been prepared by crosslinking with redox reactive degradable crosslinkers usually containing hydrazone, ketal, acetal, and disulfide bonds. Li et al. reported disulfide core‐crosslinked nanoparticles based on dextran‐lipoic acid derivatives for triggering intracellular DOX release(Figure 5A).^78^ Talelli et al. prepared biodegradable polymeric micelles composed of poly(ethylene glycol)‐b‐poly[N‐(2‐hydroxypropyl) methacrylamide‐lactate] (mPEG‐b‐p(HPMAm‐Lacn)) diblock copolymers that, upon IV administration, improved blood circulation. Covalent conjugation of a DOX methacrylamide derivative (DOX‐MA) through free radical polymerization was performed in a micellar core. This structure enables hydrolysis at the desired pH, leading to sustained drug release under acidic conditions (tumor microenvironment). Covalent entrapment of approximately 30–40% of the drug was performed in micelles, and the resulting average diameter was 80 nm. Complete release occurs after 24 h at pH ∼5, while meagre 5% release occurs at pH 7.4 (Figure 5B). Notably, in vivo studies reveal that these core‐crosslinked pH‐sensitive DOX prodrug micelles led to better antitumor activities in B16F10‐bearing mice in comparison to free DOX and micelles, showing prolonged survival compared to the group treated with free drug (Figure 5C).^79^

#### 2.1.6 Nanoparticles

Recently, nanomaterials have been broadly used in medicine since they can be engineered for the specific delivery of chemotherapeutics at the target site with reduced toxicity.^80^, ^81^ Nanoparticles combining both active and passive targeting methods increase the concentration of drugs in cancer cells without affecting normal cells. Once they enter the cell, they bind to specific receptors via endocytosis. Nanoparticles that are usually employed in drug delivery are submicron‐sized particles (1–100 nm), generally composed of polymers (micelles and dendrimers), liposomes, and organometallic compounds.^82^ Polymeric nanoparticles are solid biocompatible materials and one of the simplest forms of nanomedicine due to their easy synthetic procedure and facile tuning in structure to obtain desired properties for improving drug release, drug distribution, and efficacy.^83^, ^84^ Over the last few decades, polymeric nanoparticles have been exclusively examined in drug delivery, such as poly(L‐lactic acid (PLA) and poly(lactide‐co‐glycolide acid (PLGA), which are clinically studied and approved by the FDA. PLGA multifunctional nanoparticles loaded with Taxol have shown chemotherapeutic activity and photothermal killing of cancerous cells both in vitro and in vivo.^85^ One‐dimensional (1‐D) nanomaterials include synthetic CNTs made from carbon‐containing graphene sheets. Functionalization of CNTs has been performed for gene and drug delivery applications since they can readily permeate through biological barriers and thereby find suitable carriers as cargo inside cells without any toxic effect.^86^, ^87^

α CD‐based pH‐responsive nanoformulations have been reported in which various acetal groups and confined acetal linkages on α CD facilitated the controlled release of the anticancer drug PTX from the system. Fabrication of blank Ac‐αCD NPs and PTX‐loaded NPs was performed using the o/w emulsion technique. By controlling the reaction parameters, nanoparticles containing PTX had a morphology and size similar to those of Ac‐αCD (Figure 5D). Hydrolysis of nanoformulations in response to pH causes release of the drug, which enhances the antitumor effect in various cancerous cells. Dose‐dependent toxicity is observed with killing efficiency with increasing dose (B16F0 cell killing after 48 h is presented in Figure 5E), which is attributed to efficient internalization of nanoparticles in tumor cells.^88^ The surface of mesoporous silica nanoparticles (MSNs) is functionalized with amino β‐CD rings having disulfide bonds where DOX can be easily entrapped inside the nanoparticles for targeted delivery to cancer cells. Functionalization of PEG with AD units at one end and with folate groups at the other end followed by its immobilization on the surface of nanoparticles through strong CD–AD complexation. Drug release from nanoparticles is triggered by acidic endosomal pH followed by disulfide cleavage in high glutathione in the cytoplasm, further promoting drug release from the vehicle. The better efficacy of drug release from nanoparticles is attributed to the combined effect of folate targeting and stimulus‐triggered release, which is very well reflected in cell killing with varying drug concentrations as a function of time.^89^ Emami et al. prepared gold nanoparticles (GNs) of 12 nm and conjugated them with DOX and anti‐PD‐L1 with combination therapy, including chemotherapy and PTT, for the treatment of colorectal cancer (schematics for each step are presented in Figure 5F). The prepared GNs possessed more affinity for PD‐L1‐overexpressing CT‐26. The antitumor effect of PD‐L1‐AuNP‐DOX on CT‐26 cells was verified through a live/dead calcein assay (Figure 5G), where cells treated with PD‐L1‐AuNP plus NIR displayed intense red fluorescence, indicating an apparent cell killing efficiency. Moreover, enhanced cell mortality was observed in PD‐L1‐AuNP‐DOX compared to NT‐AuNP‐DOX or free DOX. The effective intracellular uptake of DOX was verified by severe apoptosis in CT‐26 cells due to ROS generation. PD‐L1‐AuNP‐DOX after irradiation with NIR prohibited cell proliferation, leading to enhanced apoptosis and cell cycle arrest. This new drug carrier, along with heat, synergistically inhibited cell growth and could be a promising nanomedicine for the treatment of PD‐L1‐overexpressing colorectal cancer.^90^

Balakrishnan et al. presented the role of GNs (3 nm) coupled with quercetin on MCF‐7 and MDA‐MB‐231 cancer cells, where this drug‐conjugated nanoparticle was found to be more efficient than the free drug, as reported for targeted drug delivery by enhancing the therapeutic effect of the drug.^91^

MSNs have a special structure with tunable pore and particle sizes, which result in a higher surface area that is facile for modification.^92^ Different approaches have been reported, where MSNs act as control drug release vehicles. Lui et al. reported a dual‐responsive drug delivery system for laryngeal carcinoma therapy where release was induced at higher temperature and low pH. Grafting of the thermo/pH‐sensitive polymer poly[(N‐isopropylacrylamide)‐co‐(methacrylic acid)] was performed onto mesoporous silica, which acted as a valve and regulated the diffusion of cargo embedded in and out of the pore channels depending upon the environmental conditions. The presence of covalent bonding with FA facilitates increased uptake of nanocarriers into HepG2 cells (with folic receptors). These thermos/pH‐responsive biocompatible nanocarriers have the potential to be used as targeted drug release systems for laryngeal carcinoma treatment.^93^ Kim et al. demonstrated interweaving of the CD gatekeepers connected disulfide unit of GSH to surface or mesoporous silica as potent technique for not only encapsulation of cargo into pore channel but also acts in response to GSH. The GSH‐induced release of DOX from the CD‐capped Si‐MPs was found to be effective against adenocarcinoma cells.^94^ Moreover, polymers based on cystamine have also been investigated for sealing the pore channels, and degradation of these crosslinked polymers in the presence of a disulfide reducing agent efficiently opens the network and releases chemotheraputics.^95^

Another method for controlled drug delivery from mesoporous silica is based on coating with a lipid bilayer. Nel and coworkers coated a custom‐designed lipid bilayer onto MSNs to codeliver Gem/PTX for pancreatic cancer therapy. In vivo experiments using IV injection of PTX/GEM‐loaded LB‐MSNs demonstrate significant shrinking of tumor volume compared to free gem‐gemcitabine (GEM) without posing any local systemic toxicity.^96^ Other inorganic carriers, such as quantum dots (QDs), magnetic nanoparticles, and mesoporous silica, are widely used in cancer treatment in a number of ways. QDs have already emerged as imaging probes, particularly for extended periods, quantitative imaging, and diagnostics.^97^ QDs, zero‐dimensional (0‐D) nanoparticles with sizes in the range of 1–10 nm, have been proven to be the brightest candidates for the targeted delivery of chemotherapeutics, actual time tracking of the intracellular course, and in vivo imaging due to their distinct physiochemical properties, such as uniform and narrow size distribution, higher surface‐to‐volume ratio, biocompatibility, and multicolor fluorescence imaging and detection.^98^ Recently, versatile QDs have been prepared and reported to be fascinating targeted drug delivery carriers for diagnosis and imaging in various cancer therapies.^98^ Over the past few years, the application of gold nanoparticles (GNs) in the biomedical field has attracted severe interest due to their inherent properties, which make them more appropriate for cancer diagnosis and treatment. The efficacy of GNs in cancer therapy relies on their ability to penetrate tumor tissues.^99^ A few important polymeric carriers with their composition and modes of administration are listed in Table 1.

**TABLE 1:** Different polymeric carriers, their composition, modes of administration, and their efficacy

Polymeric carriers	Composition of material	Mode of adminstration	Efficiency of carrier	References
Micelle	(H40‐P(LA‐DOX)‐b‐PEG‐OH/FA)	–	Enhance cellular uptake, cytotoxicity due to the folate‐receptor‐mediated endocytosis, and higher killing of 4T1 tumor cells.	^76^
	NK 105, PEG‐b‐poly(aspartate‐4‐phenyl‐1‐butanolate) for PTX delivery	Intravenous	Importantly strong antitumor effect on a human colorectal cancer cell line HT‐29 xenograft due to the improved assemblage of chemotherapeutic at tumor site.	^100^
Liposomes	Phosphatidyl choline, cholesterol, and ethanol for melphalan	Combination therapy	Enhanced in vivo efficacy in combination with hyperthermia in C57B1/6 mice‐bearing B16F10 melanoma with inhibition of tumor growth.	^101^
	Estrogen receptor (ER) targeted pH‐sensitive liposome for DOX delivery	Intravenous	Estrone anchored pH‐sensitive liposomes enhanced intarcellular uptake of DOX and also inhibited in vivo tumor growth.	^102^
Dendrimers	PAMAM conjugated to folic acid and MTX	Intravenous	These folate‐conjugated nanoparticles concentrated in the KB tumor cells and liver tissue. Targeting methotrexate increased its antitumor activity.	^103^
	PAMAM‐conjugated with cis platin	Intraperitonial and intravenous	Dendrimer‐Pt given i.p/i.v. showed enhanced antitumor activity approximately 50‐fold increase against B16F10 due to dedrimer‐Pt accumulation in solid tumor tissue by the EPR.	^104^
Hydrogels	Bi(mPEG‐PLGA)−Pt(IV) (PtGel)	Intratumoral	Single intratumoral injection of this hydrogel in ovarian tumor showed excellent in vivo anticancer efficacy and significantly reduced side effects.	^105^
	PNAm‐PDAAu‐DOX	Combined therapy (photothermal and injection)	(SPN) hydrogels prevented the recurrence of breast cancer, and can be tracked by computed tomography (CT) imaging due to loaded AuNPs.	^106^
Polymer drug conjugates	Poly‐R‐(L‐glutamic acid) (PG) conjugates of CPT	Intraperitonial	Enhanced efficiency of PG‐gly‐CPT in the HT‐29 colon and NCI‐H460 lung carcinoma after increased loading of CPT.	^107^
	PLGA‐GEM	Subcutaneous	This formulation showed the strongest antitumor effect, likely due to the proper “release” of GemC18 from the injection site.	^108^

### 2.2 Dimensional effect of particles on chemotherapy (inorganic nanocarriers)

The properties of particles strongly depend on the shape and size, and the specific properties include drug delivery for disease control. By confining the different dimensions, the particles are classified into 0‐D, 1‐D, two‐dimensional (2‐D), and 3‐D particles. The usefulness of the particles with different dimensionalities is discussed separately.

#### 2.2.1 Carbon dots

Zero‐dimensional nanoparticles, such as QD carbon/heavy metals with sizes of 1–10 nm, have emerged as one of the most favorable nanoparticles for targeted drug delivery systems, where live monitoring of intracellular processes and in vivo imaging are performed owing to their inherent unique physicochemical properties, such as a higher surface‐to‐volume ratio, nontoxicity, highly tunable luminescence, and other properties.^98^ Luminescent carbon dots (C‐Dots) have recently attracted considerable attention owing to their tremendous potential in biology as labeling/imaging agents, photocatalysts, sensors, and building units for prospective nanocarriers.^109^, ^110^ Compared to conventional metal‐based QDs, these carbon‐based nanoparticles possess excellent photostability, biocompatibility, solubility in water, and low toxicity.^111^ Zhang et al. prepared a multifunctional drug delivery system triggered by the pH of the system, where they anchored C‐Dots onto heparin via chemical bonding and then loaded the potent anticancer drug DOX via electrostatic interactions between the drug and CDs‐hep. The developed CD‐hep/DOX system exhibits better stability and pH‐responsive drug release. The prepared system displays higher toxicity against cancer cells with better therapeutic efficacy as measured through the MTT assay. Furthermore, the internalization of these materials by A549 cells was studied using laser scanning confocal microscopy. Zhou et al. developed a biocompatible system for rational on‐demand delivery and cellular imaging comprising C‐Dots capped on the surface of MSPs. Transmission electron microscope (TEM) was used to study the distribution of C‐Dots on the surfaces of NH_2_‐MSP (Figure S1A). The C‐Dots@MSPs were used for in vivo imaging after SC injection due to their excellent optical property for cell labeling. The corresponding injection areas are displayed clearly through the PL signal, and the imaging of SC tissue is found to be effective (Figure S1B), suggesting the potential of C‐Dots@ MSPs. The drug release studies in Figure S1C showed the amount of DOX released over time at pH values of 5.0 and 7.4. Primarily, a lower amount of DOX was released at pH 7.4, while at pH 5.0, there was a steady release over 8 h (Figure S1D). The cellular uptake of C‐Dots@MSPs‐DOX in HeLa cells was investigated, where C‐Dots@MSPs were remarkably internalized into the cells and distributed mainly in the lysosomes.^112^

#### 2.2.2 Carbon nanotubes

CNTs are synthetic 1‐D nanomaterials derived from carbon that contain rolled graphene sheet rings built from sp^2^ hybridized carbon atoms into hollow tubes.^113^ CNTs are made of carbon cylinders where benzene rings are the basic constituents, and CNTs have recently attracted significant attention for their application in the biomedical field. The application of CNTs as drug carriers at the site of interest has been one of the prime areas of research by different groups, which is attributed to their characteristic properties, including their distinctive chemical, physical, and biological properties, shape, hollow structure, and easy surface modification. Modification of CNTs with polymers is basically done via physical interactions or chemical connectivity, usually covalent bonding. Two commonly used approaches for the covalent bonding of polymers onto CNT surfaces are grafting through or grafting from the method. Adeli et al. reported CNTs grafted with a hyperbranched polymer through a grafting method. The developed hybrid materials are hemocompatible and show cytotoxicity toward HT1080 cells.^114^ Their shape enables them to easily enter the cells via different techniques, including passive diffusion or endocytosis. CNTs first attach to the cell surface and then are engulfed by the cell membrane. Li et al synthesized P‐gp antibody‐functionalized CNTs incorporated with DOX and revealed the cytotoxicity caused by the materials to MDR leukemic K562 cells, which is more efficient than DOX in pure form.^115^ In another study by Dhar et al., a platinum IV complex containing FA uniquely targets folate receptor tumor cells, but more efficient and targeted delivery of platinum‐based chemotherapeutics was observed when it was conjugated with CNTs.^116^ Furthermore, the larger inner diameter of CNTs is more facile for drug loading. Cisplatin and DOX are loaded into mildly oxidized multiwalled carbon nanotubes (MWCNTs) with large inner diameters. Incorporation of PEG and FA is performed onto the nanocomposites to obstruct the release of chemotherapeutics from the inner zone of the nanotubes. Thus, these nanocomposites exhibit higher cytotoxicity to cancer cells than pure MWCNTs.^117^ Qin et al. reported thermo and pH‐sensitive nanogels based on amphiphilic chitosan derivative‐coated single‐wall CNTs encapsulated in a thermo/pH‐responsive nanogel (CS/PNIPAAm@CNT) loaded with DOX. DOX release from these nanocomposites is triggered by temperature and pH. Faster release is observed at higher temperatures at 40°C than at 25°C. Similarly, more DOX release occurs at pH 5.0 than at to pH 7.4 (Figure S1E). Without laser irradiation, less toxicity is observed, which means that DOX‐CS/PNIPAAm@CNTs serve as a matrix reservoir for DOX, while after NIR irradiation for 10 min, DOX‐CS/PNIPAAm@CNTs exhibit enhanced cell killing effects (Figure S1F), which is further verified by better cell internalization after 24 h of incubation, as shown from the confocal microscopy images (Figure S1G). Finally, the combined effect of NIR irradiation for thermal effects and an acidic environment considerably enhanced DOX release.^118^

#### 2.2.3 Graphene

Generally, graphene‐based nanomaterials are classified as 2‐D materials and are usually available in the form of GO and reduced GO (rGO). These nanomaterials are of significant use in the biomedical field due to their variable and controllable physical as well as chemical properties, biocompatible nature, and easy availability.^119^ Several reports on the administration of GO and rGO in drug delivery, cell targeting, biosensing, and bioimaging are well presented in the literature, also conferring them as potential agents for targeted cancer therapy.^120^, ^121^, ^122^ One of the major approaches for targeted drug delivery in cancer is its modification of GO/rGO with suitable targeting ligands. Conjugation of GO (GO‐COOH) is prepared with hydroxypropyl β−CD for targeted release of PTX. This delivery system exhibits a higher PTX loading capacity with better aqueous stability. The release of a drug is pH dependent, and its improved blood compatibility is well known.^123^ Similarly, in another work by Yang et al., GO was conjugated with carboxymethyl chitosan and hyaluronic acid (HA) for DOX delivery via noncovalent π−π interactions, and the schematic is presented in Figure S1H. Furthermore, the functional groups and chemical connectivities are proven using Fourier Transform Infrared Spectroscopy, where GO spectra display all the characteristic peaks. Upon coupling with chitosan, NH stretching peaks become prominent along with new amide peaks, indicating GO‐CMC formation (Figure S1I). The morphologies of GO, GO‐CMC, and GO‐CMC‐FI‐HA display a lamellar structure with no aggregation, indicating surface modification of GO without disturbing its intrinsic structure. The loading capacity of DOX was found to be 95% with faster release under acidic conditions (pH of tumor microenvironment) than at normal physiological pH 7.4 (Figure S1J). These conjugated materials specifically target cancer cells by attacking overexpressed CD44 receptors in cancer cells.^124^ Covalent bonding of GO/rGo with drugs is another approach for targeted release. Wojtoniszak et al. studied drug release and its anticancer behavior through amide linkages loaded with MTX on GO.^125^

#### 2.2.4 Layered double hydroxides

Layered double hydroxides (LDHs), one of the important inorganic 2‐D carriers, have numerous captivating features for their application as drug delivery agents, especially for negatively charged drugs. The properties of LDH include capacity for anion exchange, biological compatibility, nontoxicity, and use as an injectable cargo carrier. The layers present in LDH possess a positive charge, which is neutralized by the presence of anions in the interlayer spacing and can easily be substituted by electronegative biomolecules, such as drugs, vitamins, DNA, and amino acids.^126^ Synthesis of LDH with various negatively charged ions, such as CO_3_^2−^, PO_4_^3‐^, and NO_3_^−^, is achieved by a coprecipitation method, and the aqueous chemotherapeutic raloxifane hydrochloride is inserted via an ion exchange method. An in vitro drug release study from drug‐intercalated LDH with different nanostructures revealed prolonged drug release, showing 100% release in 42 h. Controlled drug release from drug‐loaded LDH has been attributed to the interactions between LDH and the drug; the stronger the interaction is, the slower the release kinetics. Furthermore, a sustained release pattern is evident from cellular studies on HeLa cells, resulting in cell growth inhibition by the cargo‐loaded LDH. Additionally, these drug‐loaded LDHs showed higher tumor suppression with no significant reduction in body weight and no considerable impairment to body organs.^127^ In another approach for attaining more significant sustained release for cancer treatment, drug‐intercalated LDHs are integrated in a polycaprolactone (PCL) matrix to develop injectable drug carriers with better curative efficacy. Prolonged release of cargo is visualized for the LDH system due to greater interactive forces between the drug and LDH, while in the case of the polymer system, the PCL matrix provides enhanced bioavailability of hydrophobic anticancerous drugs. These nanohybrids not only suppressed the fast release of the drug but also maintained hydrophilic and hydrophobic balance, resulting in prolonged release for 4 days. The cellular uptake efficacy for these drug carriers was studied by labeling the drug and LDH with a fluorescent dye, rhodamine B. Interestingly, sufficient fluorescence was observed in the polymer nanohybrid (PN‐RhdB: rhodamine B‐tagged nitrate (NO_3_^−^) LDH and its subsequent housing in the PCL matrix). Different inorganic carriers used for chemotherapeutic delivery with their composition and modes of administration are summarized in Table 2.

**TABLE 2:** Various inorganic nanocarriers, their composition, and mode of treatment

Inorganic carriers	Composition	Mode of administration	Efficiency	References
Nanoparticles	GSH‐responsive HDMP	Chemotherapy and PDT	In vivo pharmacoimaging by PET imaging demonstrated that HDMP NPs significantly improved drug delivery to tumor.	^128^
Carbon nanotube	SWNT FA‐SWNT	Intraperitonial and photothermal	FA‐SWNT efficaciously improved the photothermal ablation of mammary carcinoma tumor cells.	^129^
	SWNT‐PTX	Intravenous	SWNT‐PTX showed higher efficacy in suppressing tumor 4T1 breast cancer due to prolonged and 10‐fold higher tumor PTX uptake by SWNT delivery (EPR).	^130^
Gold nanoparticle	Polyethylene glycolcoated gold nanorods (PEG‐NRs)	Photothermal	Localized plasmonic heating of ovarian tumors enhances accumulation therapeutic agents doxorubicin liposomes in this orthotopic tumor model.	^131^
	Pegylated silica‐core gold nanoshells (pSGNs)	Hyperthermic intraperitoneal	The gold nanoshells conjugated with anti‐CD47 antibodies efficiently killed cancer cells, and reduced the required amount and duration of NIR irradiation.	^132^
Mesoporous silica	Dox‐UCNP@mSiO2‐azo	Combination (IV and PTT)	NaYF4: TmYb UCNPs with azo‐modified mesoporous silica caused controlled release of drug by modulating the intensity and/or time duration of NIR light irradiation.	^133^
Quantum dots	(BPQDS‐PEG‐FA/DOX)	PTT	The BPQDs‐based drug delivery system exhibited pH and photoresponsive release properties, and excellent photothermal performance was also demonstrated in vivo.	^134^

## 3 MODE OF ADMINISTRATION OR DIFFERENT THERAPIES FOR CANCER TREATMENT

**FIGURE 6:**
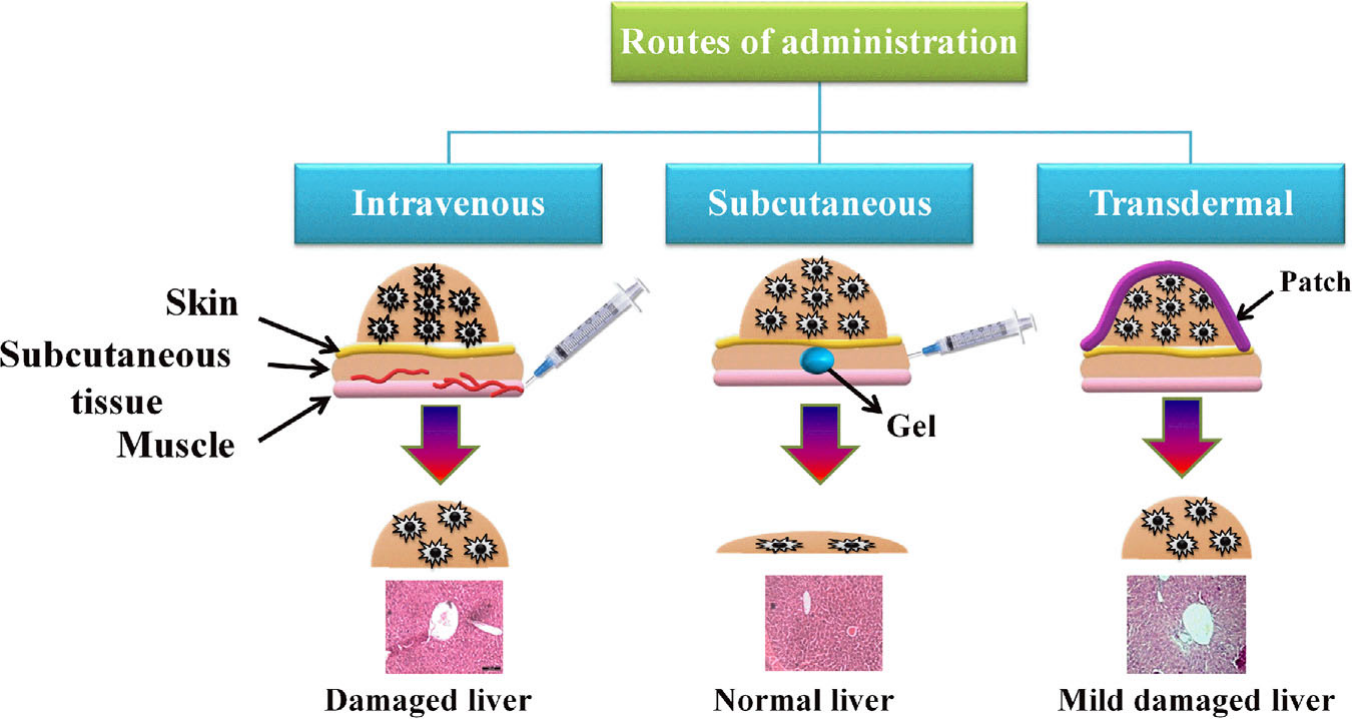
Schematic for different routes of administration of drug carriers in the treatment of cancer. Images of livers are reproduced with permission from Refs. ^51^ and ^65^ Copyright 2019 Elsevier, Copyright 2019 American Chemical Society

The development of nanomedicines for controlled drug release has continuously increased and has gained interest over the past several years due to their multiple applications, which include targeted drug delivery toward specific organs or tissues, controlled release, enhanced cellular uptake, and improved pharmacokinetic effects. The nanocarriers discussed in the above section have been explored in different routes for their administration, mostly IV, oral, transdermal, SC, and ocular (Figure 6), as well as in different therapeutic methods used for cancer treatment, including PTT, PDT, radiation therapy, hadron therapy, chemodynamic therapy, gas therapy, and immune therapy.

### 3.1 Intravenous administration

Parenteral administration (injections or infusions) remains the customary course for chemotherapy since the whole dispensed dose is immediately circulated within the body fluid and is a well‐developed methodology.^135^ This route of administration of the drug completely depends on its pharmacological properties (e.g., absorption, metabolism, and half‐life), and the bioavailability of most chemotherapeutics is very low; thus, their common route of administration is IV injection. In the IV route, drugs are usually discharged into the subclavian vein or alternatively into the cephalic vein in the arm or the femoral vein in the groin. The advantage of delivering drug in the subclavian vein is that it leads directly to the heart; as a result, rapid distribution of the drug occurs through systemic circulation.^136^

IV administration is commonly used, and infact, this is the prime administration route for cancer therapy.^137^ Among the nanocarriers for chemotherapy, both polymeric and liposomal carriers have found success clinically, and they are primarily administered via IV.^138^ Improved pharmacokinetic effects and reduced drug toxicity are important advantages of using chemotherapeutic nanocarriers. Moreover, the EPR effect is another benefit of IV administration. The mechanism is based on the leaky blood vasculature around tumor tissues, allowing easy passage of these nanocarriers and employing their effects.^139^

To promote EPR, a coating of nanocarriers is often designed with a hydrophilic or neutral polymer that prevents protein aggregation on the nanocarrier surface and reduces clearance in the reticuloendothelial system.^140^ Kim et al. developed a new low molecular weight, conducive, ecofriendly, amphiphilic diblock copolymer of monomethoxy poly(ethylene glycol)‐block‐poly(D,L‐lactide) (mPEG‐PDLLA) for improving the treatment efficiency in melanoma mice by IV administration of PTX. From the results exhibited by the biodegradable polymeric micelles embedded in PTX, the therapeutic potential was enhanced toward a variety of solid tumors and can be clinically used against human solid tumor treatment.^141^

**FIGURE 7:**
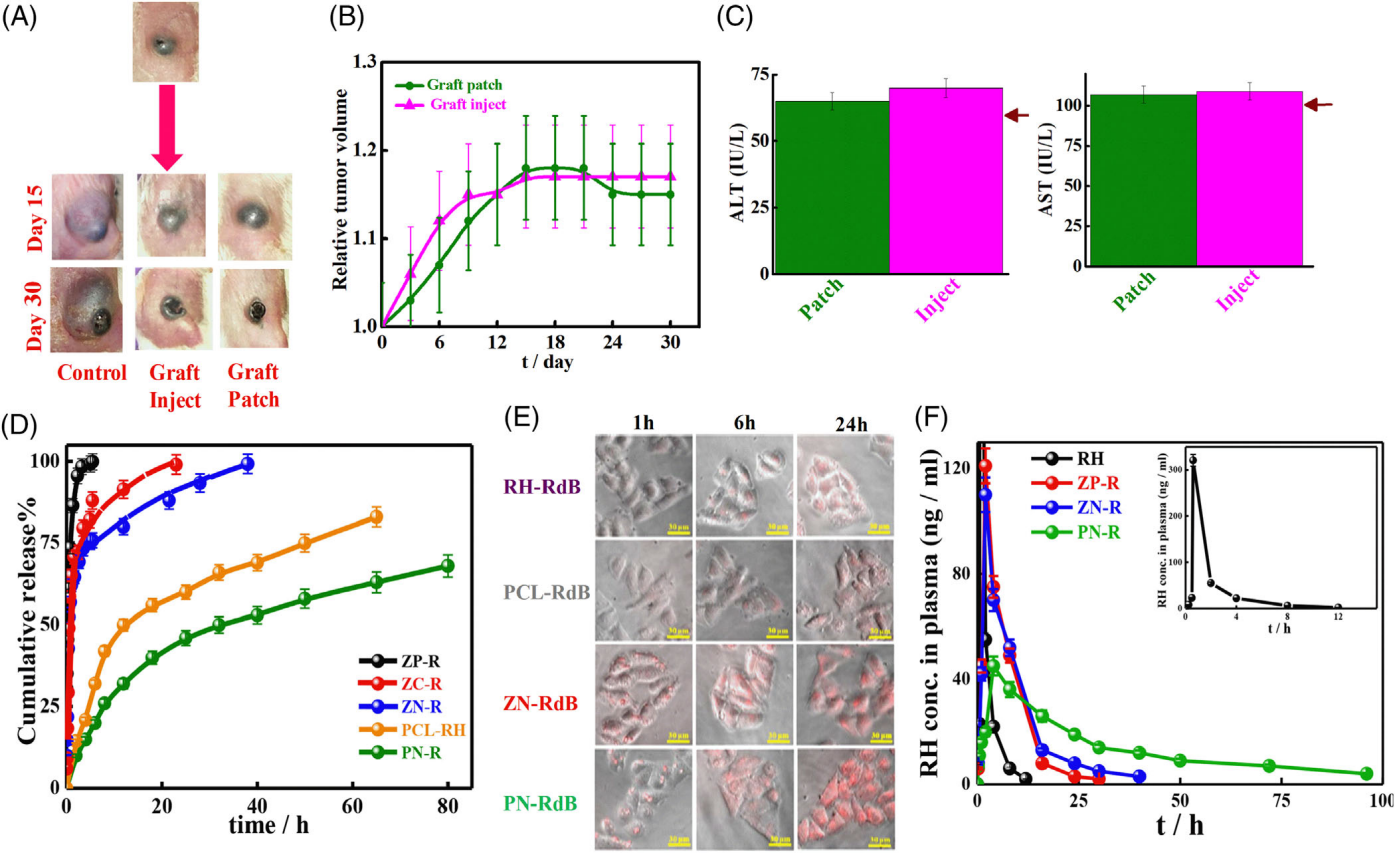
Intravenous mode of administration by graft copolymers and nanoparticles: comparison of efficacy of graft patch versus conventional graft inject system; (A) images of mice after 15 and 30 days of treatment with patch and injected systems; (B) relative changes in tumor volume with time; (C) biochemical parameters, ALT and AST. The arrows indicate the corresponding values in healthy mice. Reproduced with permission from Ref. ^51^ Copyright 2019 Elsevier. (D) In vitro drug release profile for raloxifene‐intercalated LDHs (ZN‐R, ZC‐R, and ZP‐R), raloxifene embedded in PCL matrix (PCL‐RH), and PCL‐coated ZN‐R (PN‐R); (E) cellular uptake by HeLa cells under different incubation times; (F) biodistribution for RH, ZN‐R, ZP‐R, and PN‐R versus time profiles after intravenous administration. Reproduced with permission from Ref. ^142^ Copyright 2018 American Chemical Society

The other system comprising polyurethane‐grafted CD copolymers is injected in solution form intravenously, and images of the tumor after 15 and 30 days of treatment are displayed in Figure 7A. After 30 days of treatment, mice treated with patch displayed a reduction in tumor volume (Figure 7B), and further, biochemical parameter analyses, especially alaninie amino transferase (ALT) and aspartate amino transferase (AST) values, were considerably increased (Figure 7C) from the normal values in mice treated with IV compared to patch, suggesting improper liver functioning.^51^ In another work by Senapati et al. developed a novel nanohybrid drug carrier where they incorporated drug‐loaded LDH into polycaprolactone (PCL) matrix to enhance the efficiency of raloxifane both in vitro and in vivo for better cancer cell killing and to mitigate adverse side effects caused to vital body organs. These developed nanohybrids restrained the fast release of raloxifine, and controlled release was observed for 4 days (Figure 7D). To determine the LDH and drug inside cells, both were labeled with fluorescence rhodamine B (RdB). Greater uptake, that is, sufficient fluorescence was observed for nanoparticles after 24 h against a lower portion of pure drug that could enter the cell in a similar time frame (Figure 7E). Furthermore, from the in vivo results after IV administration, almost 100% release of pure drug was observed after 8 h, while for LDH nanohybrids ZP‐R and ZN‐R, controlled release was obtained up to 30 and 40 h, respectively (Figure 7F). The efficacy of sustained release from these novel drug carriers in the bloodstream has been visualized from animal model studies, and their nontoxicity toward sensitive body organs has been demonstrated from histopathological studies displaying normal healthy liver and other body parts, such as the kidney, when treated with LDH polymer nanohybrids against damaged liver using pure raloxifine.^142^

He et al. prepared trimethyl chitosan (TMC)‐based drug conjugates modified with FA for targeted IV and oral delivery of PTX. Due to the amphiphilic nature of these drug conjugates (TMC‐PTX and FA‐TMC‐PTX), they self‐assembled into spherical nanoparticles with an average size of 170–187 nm. In vitro drug release studies of these conjugates demonstrate sustained release of PTX, which depends on the pH of the release medium. These drug conjugates enhance mucoadhesion compared to pure TMC, thus encouraging the ex vivo intestinal movement of PTX, illustrating its favorable safety in blood. From pharmacology and biodistribution studies, prolonged retention of drugs from these conjugates in blood and their improved accumulation in tumor tissue enhance their tumor inhibiting efficacy after IV injection in comparison to pure PTX injection.^143^ In another report, Wang et al. prepared covalent organic crosslinked polymeric (COPs) networks composed of organic molecules. A novel pH‐sensitive COP based on acryloyl meso‐tetra(p‐hydroxyphenyl) porphine (acryloyl‐THPP) was used as a PDT agent for constructing a hydrophobic core to react with 4,4′‐trimethylene dipiperidine (TMPD) to form pH‐responsive crosslinked biodegradable β‐amino esters (BAEs). Finally, biocompatible PEG is used to form a protective hydrophilic shell. Owing to the porous structure, encapsulation of a potent anticancer drug (DOX) is developed in these pH‐sensitive nanostructures. On IV administration of THPP‐BAE‐PEG/DOX to tumor tissue, the weakly acidic tumor microenvironment initiated hydrolysis of BAEs, leading to the dissociation of nanostructures, and the release of encapsulated drug occurred from THPP‐BAE‐PEG/DOX.^144^ A distinctive class of metallodrugs containing a nonsteroidal anti‐inflammatory drug (NSAID), Ru_2_(NSAID), has been prepared and exhibited anticancer effects in vitro and in vivo in glioma cells.^145^ The considerable benefit of IV administration is the direct and immediate release of products into blood circulation,^146^ which improves bioavailability. The problems related to therapeutic degradation are lowered in IV administration, permitting more effective delivery of sensitive carriers.^147^

### 3.2 Subcutaneous delivery

Delivery of cargo into the interstitial area beneath the epidermis is performed subcutaneously. Glycosaminoglycan and collagen fibers are negatively charged tissues present in these interstitial areas that contribute to steady and slow absorption of molecules to reduce blood flow.^148^ The SC mode of drug delivery is mostly used for nanoparticle delivery,^149^ while traditional vaccines are administered via the intramuscular (IM) route.^150^ Through the SC mode of delivery, the delivery of nanoparticles is substantially increased in draining lymph nodes, effective uptake of nanodiscs by antigen‐presenting cells, and generation of a seven‐fold higher frequency of neoantigen‐specific T cells when compared with the IM route. The prepared nanodiscs together with anti‐PD‐1 and anti‐CTLA‐4 IgG therapy in melanoma tumor‐bearing mice displayed significant antitumor effects, resulting in the extinction of tumors induced in ∼60% of animals.^151^ Lee et al. synthesized a triblock copolymer where functionalization of vitamin E was made with polycarbonate and polyethylene glycol, forming physically crosslinked injectable hydrogels for delivery of herceptin in a controlled manner. The antitumor specificity and efficacy of hydrogels were studied in normal and breast cancer cell lines at different HER2 expression levels. Hydrogels loaded with Herceptin showed specificity toward HER2‐overexpressing cancer cells and were toxic, very similar to Herceptin solution. Analysis of the biocompatibility and biodegradability of hydrogels was performed by SC injection into mice, revealing no inflammation within 6 weeks through histological studies.^152^ A single dose of Herceptin‐loaded hydrogels administered subcutaneously in BT474‐bearing mice showed awesome retention of antibody inside the tumor, ultimately leading to collapsed tumor size by 77% at 28 days against IV and SC delivery of pure Herceptin solution.^152^ Vitamin E‐ and PEG‐based triblock copolymer biodegradable hydrogels are used for the delivery of OVA, a model antigen hepatitis B drug. Its effective delivery would result in a superior immune response against cancer. This triblock copolymer exhibits a flower‐like arrangement where PEG is exposed to aqueous solution, forming a hydrophobic core at higher polymer concentrations and ultimately forming hydrogels. Encapsulation of OVA occurs during gel formation. OVA‐loaded gel containing aluminum‐based adjuvant restricts tumor occurrence, and only 2 out of 10 mice develop solid tumors with significantly smaller tumor sizes, revealing its efficacy for sustained delivery of cargo.^153^ SC injection of polyurethane‐grafted dextrin hydrogels has been reported without any adverse effects on body organs and improving mouse survival, thereby making this brush polymer hydrogel a promising drug carrier.^154^ SC administration is typically convenient compared to IV and IM administration. Moreover, it is less painful, less time‐consuming, and offers better patient compliance.^155^ Additionally, nanocarrier formulations administered subcutaneously provide protection to chemotherapy, enhancing extended release for a longer duration and thereby reducing the number of doses, better efficiency, and targeted delivery of cargo.^156^

### 3.3 Transdermal delivery

The transdermal mode of delivery refers to the administration of a drug through the skin and the attainment of systemic treatment for clinical applications. It has become the third largest mode of delivery systems after oral and injection administration. It offers several advantages, such as a facile administration route of the drug, can lessen the toxic effects of the drug, and can reduce fluctuations in drug concentrations in the bloodstream. However, its efficiency is low due to the stratum corneum, which is the largest barrier in the transportation of drugs or other biological materials. Thus, it is necessary to find suitable methods that could enhance the transdermal permeation of drugs. The properties of the transdermal patch could be altered, which allows easy diffusion through the skin. Chemical methods mostly include the addition of a permeation enhancer^157^ that interacts with the material and enhances skin permeability.^158^ The addition of biopeptides increases skin permeation by conjugating with model drugs. Other physical methods, such as ultrasound methods, microinjection, and intradermal injections, are utilized for better permeation. Recent technologies in which nanocarriers and patches are applied have enlarged the range of dermal routes of systemic drug delivery systems. Transdermal delivery formulations that are generally employed include creams, lotions, sprays, ointments, and patches (requiring permeation enhancers).^159^ The encapsulation of cargo in nanocarriers not only enhances their penetration and absorption rate but also provides the shielding effect from early degradation and, most importantly, controls release.^160^ Two different modes of transdermal administration are discussed, that is, microneedles and transdermal patches for cancer therapy.

#### 3.3.1 Microneedles

Microneedle‐based drug delivery systems have become an outstanding approach for transdermal administration in recent years.^161^, ^162^ Microneedles are usually four types of solid microneedles,^163^ coated microneedles,^164^ hollow microneedles,^165^ and dissolving microneedles.^166^ They are composed of various materials, such as metals,^167^ inorganic,^168^ and polymeric materials.^169^ Recently, microneedles have been used in the delivery of drugs,^170^ genes,^171^ proteins,^172^ RNA,^173^ and vaccines.^174^ Combined use of microneedles and other nanomedicines has been used in cancer treatment, diagnosis,^64^ and immunotherapy.^175^ The application of metal microneedles for drug delivery limits their use due to their shape and size, and they may break inside the skin, which may lead to safety issues. Inorganic microneedles possess similar characteristics and properties as metal microneedles; thus, their biocompatible nature and brittleness are the main concerns that limit their applications. Polymer‐based microneedles are the most promising materials for drug delivery. A large number of polymeric materials are used, such as PLGA,^176^ PLA,^177^ PCL,^178^ HA,^179^ poly(vinyl alcohol),^180^ carboxy methyl cellulose,^181^ chitosan,^182^ and many more. For tumor therapy, transdermal drug delivery systems are superior administration, since transdermal drug delivery systems increase the localized drug concentration and minimize the side effects caused to vital organs, such as the liver. Hao et al. prepared a combined system of NIR‐responsive PEGylated gold nanorod (GNR‐PEG)‐coated poly(L‐lactide) microneedles (GNRPEG@MNs) and DTX‐loaded MPEG‐PDLLA micelles for human epidermoid cancer therapy. GNR‐PEG‐absorbed PLLA microneedle not only possesses better skin insertion ability but also acts as an efficient heat transfer agent at the tumor site at approximately 50°C, which is sufficient for tumor removal.^183^ Jain et al. coated 5 aminolevulinic (5‐ALA) acid on microneedle patches (57 microneedles) prepared via a microprecision dip coater to improve dermal delivery. Once applied dermally, 5‐ALA is converted naturally by the cells/tissues into the photosensitizer protoporphyrin IX. Coated microneedles were effective, even at lower doses of 5‐ALA, in restricting the growth of SC tumors compared to typical cream formulations of 5‐ALA, which were ineffective in suppressing tumor growth in porcine skin.^184^ An encouraging immunotherapy strategy via a transdermal microneedle patch was reported by Ye et al., where the tumor lysate of B16F10 cells containing melanin was loaded in a microneedle patch. The temperature of the melanin‐loaded microneedle patch increased to 40°C under treatment with near‐infrared (NIR) light, which enhanced the tumor antigen uptake ability. In vivo results suggest that the microneedle patch increased the survival rate of mice along with improved immune responses.^185^

#### 3.3.2 Patch

Immunotherapies have become important for skin cancer therapy. Microneedle patches have been prepared for tumor immunotherapy. In one of the reports, a microneedle patch was used for the controlled release of α‐PD1 under physiological conditions. The microneedle consists of HA incorporated in pH‐sensitive dextran nanoparticles, where α‐PD1 and GOx are absorbed, converting blood glucose to gluconic acid. These nanoparticles undergo self‐dissociation, resulting in α‐PD1 release in an acidic environment. Administration of HA dissolved in microneedle patches effectively penetrates the skin, and mice‐bearing melanoma tumors demonstrate exceptional antitumor efficacy by exclusively increasing the retention time of α‐PD1 at the tumor site, which ultimately results in cancer cell killing.^186^

**FIGURE 8:**
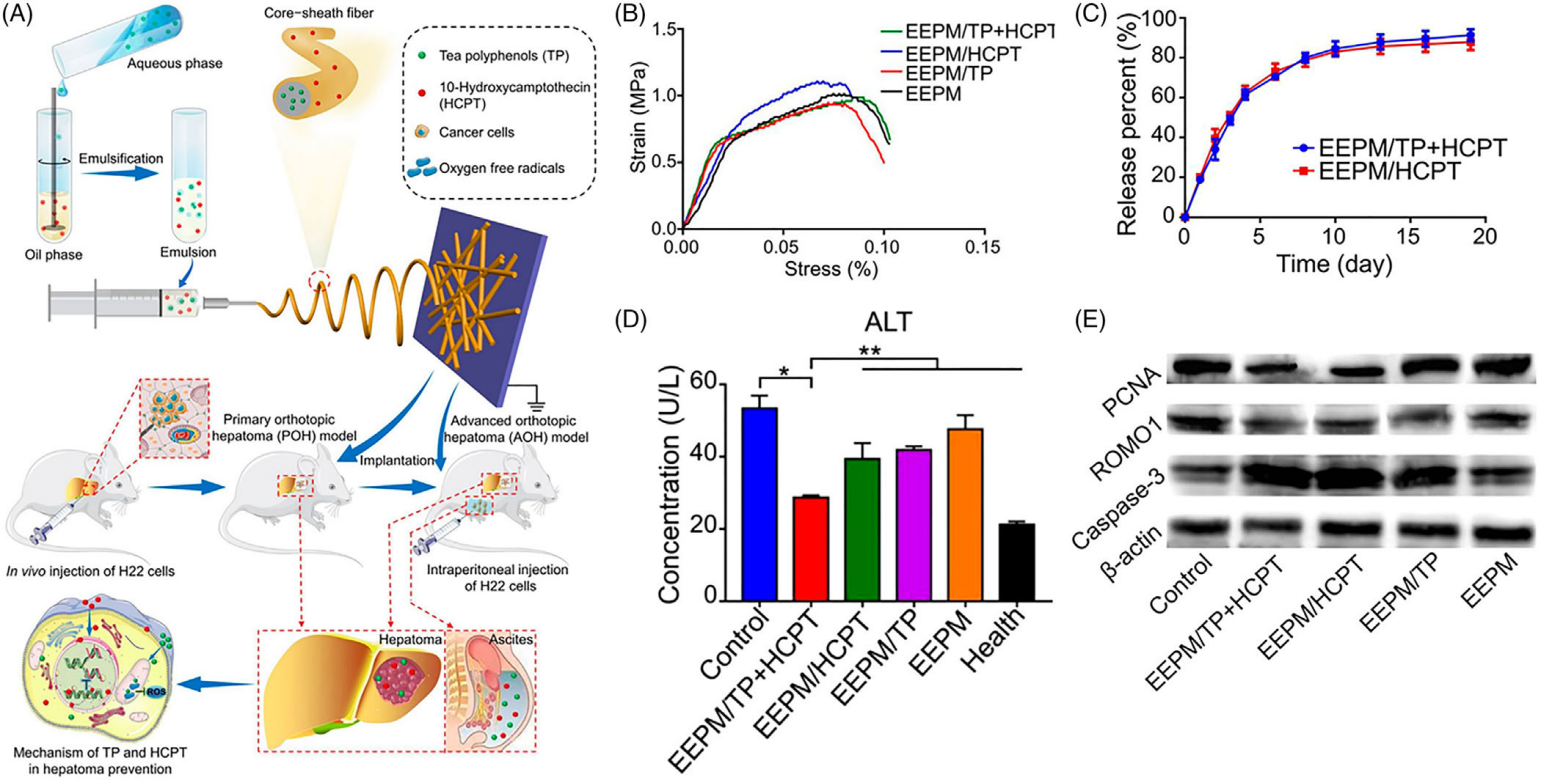
Transdermal delivery through nanofiber patch: (A) schematic illustration of preparation of HCPT and TP coloaded emulsion electrospun membrane (EEPM/TP+HCPT), construction of POH and AOH models, and synergy mechanism of EEPM/TP+HCPT against hepatoma; (B) stress starin curve for emulsion electrospun membrane; (C) HCPT release behaviors of EEPM/HCPT and EEPM/TP+HCPT in PBS; (D) biochemical analyses of ALT in POH model; and (E) western blot analyses of expressed protein levels of PCNA, ROMO1, and caspase‐3 in the tumor tissues. Reproduced with permission from Ref. ^188^ Copyright 2018 American Chemical Society

Nanofibrous patches composed of PCL and gelatin blends in different ratios were prepared through the electrospinning method for higher loading of piperine, which showed anticancerous activity along with antibacterial, anti‐inflammatory, and antioxidant properties. In vitro drug release studies demonstrate sustained release patterns, and 50% drug release is observed in 3 days from these fibrous blends. Cell viability and cell growth of HeLa cells and MCF‐7 cells were reduced after treatment with piperine‐eluting nanomats, suggesting their anticancerous activity. Flow cytometry studies reveal the generation of ROS, which leads to the killing of cancer cells.^187^ Li et al. developed a localized drug delivery carrier in the form of a patch prepared through emulsion electrospinning composed of hydrophobic hydroxycamptothecin (HCPT) and hydrophilic tea polyphenol (TP), forming a shell, while the nanofiber of methoxy poly(ethylene glycol)‐block‐poly(lactide‐co‐glycolide) (mPEG‐b‐PLGA) was used as the core (Figure 8A). HCPT is used to control the maturation and cancerous transformation of hepatoma, while TP is aimed at diminishing the degree of O_2_ free radicals, thus preventing the penetration and metastasis of cancer cells because of this core shell architecture. HCPT and TP exhibit prolonged and successive release, initially with HCPT followed by TP. Different nanofibers made of EEPM, EEPM/HCPT, EEPM/TP, and EEPM/TP+HCPT represent the blank emulsion‐electrospun membrane and the membranes loaded with HCPT, TP, and TP+HCPT, respectively, and the surface morphology of all the nanofibers is smooth and homogenous with drug crystals at the surface, as revealed from scanning electron microscope (SEM) studies. Furthermore, the mechanical properties of all the nanofibers possess similar mechanical strengths (Figure 8B). The cumulative HCPT release profiles for EEPM/HCPT and EEPM/TP+HCPT are presented in Figure 8C, showing no considerable difference in HCPT release from both systems, and approximately 62% release was observed. The efficiency of EEPM/TP+HCPT against primary orthotopic and advanced orthotopic hepatoma (POH and AOH) was studied through in vivo experiments, where electrospun patches were implanted and tissue analysis was performed. Reduced tumor volume and normal liver histology were observed in the EEPM/TP+HCPT‐treated group. Furthermore, ALT values, from biochemical analysis, in the POH model were increased compared with those of healthy mice (Figure 8D). Western blotting was used to analyze the protein levels of PCNA, ROMO1, and caspase‐3 in tumor tissues in the POH model, revealing the inhibition of PCNA expression by the EEPM/TP+HCPT group (Figure 8E). The potential superiority of the core‐sheath structured nanofiber membrane is evident in the localized treatment of both primary and advanced orthotopic hepatoma.^188^

A dermal patch comprised of polyurethane grafted CD loaded with cargo was recently reported by the authors.^44^ A sustained drug release pattern is obtained from grafted systems against burst release in pure drug. The release is triggered by grafted polyurethane chains, which create a tortuous path for the drug to come out. The efficacy is well reflected from the cellular studies where 75% killing efficiency obtained from grafted systems is due to sustained release. On the other hand, meagre killing from pure drug is reported owing to its burst release. In vivo studies on melanoma mice comparing the relative efficacy of patch against conventional injection. Graft copolymer patches are administered at the tumor site and significantly reduce the tumor volume as opposed to free drug. In addition, the body weight of mice indicates the increasing trend signifying its better health for the mice group treated with grafted patches. Biochemical parameters of liver and renal function tests indicated elevated values in the pure drug‐treated group, while the values were normal for the mice treated with the patch. Biochemical parameters are further corroborated from histopathological images of severely damaged liver of the mice treated with pure drug against the normal liver morphology observed in mice treated with patch.^51^ Kumar et al. reported electrospun PLA‐based nanofiber scaffolds to control drug release in a sustained manner. The slow release and better biocompatibility of the nanofiber scaffold made it a suitable biomaterial for in vivo applications. The use of scaffolds as patches on melanoma mice over the tumor site results in a considerable reduction in tumor volume without any toxic side effects on vital body organs.^189^ Apart from several advantages of dermal delivery, there are some associated barriers, such as incursion and enzymatic barriers. Moreover, for transdermal delivery, only a few chemotherapeutics are suitable, and higher molecular weight polymers have difficulty penetrating into the skin bed. Some formulations, such as patches, may lead to patient discomfort, such as annoyance and irritation. The therapeutic is released slowly due to slow absorption in dermal delivery, so it is not acceptable where quick action of therapeutics is needed.^190^

### 3.4 Photothermal therapy

PTT is a recently developed and encouraging therapeutic strategy that has been developed as a safe and localized treatment technique, wherein NIR light is used to resonate the electrons of nanoparticles and the heat liberated helps in ablating the malignant tumor. This controlled and confined heating of cancer cells leaves almost no chance to develop resistance and reduce the probability of reappearance.^191^ Usually, the materials used in PTT are of nanometer size and absorb NIR light, which penetrates living tissues without causing adverse side effects. Absorption of light by these nanosized materials is converted into heat very quickly, which in turn induces apoptosis in cancer cells, bypassing the flaws of hyperthermia PTT.^192^ To date, numerous photothermal agents have been fabricated, but most of them are nondegradable, unable to deliver standalone PTT without the assistance of chemotherapeutics, and have not been tested for long‐term biocompatibility.

**FIGURE 9:**
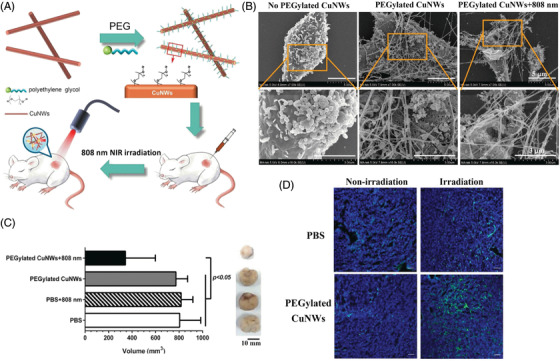
Different photothermal agents for cancer therapy: (A) schematic presentation of PEGylated CuNWs as a potential photothermal agent for cancer treatment; (B) SEM image of CT26 cells after 1 h of interaction with PEGylated CuNWs (2.5 mg/ml), with and without subsequent NIR laser irradiation for 6 min; (C) graph showing tumor volume of mice after treatment with PEGylated copper nanowires and photothermal excursion; and (D) tumor necrosis analysis through HMGB1‐specific immunostaining. Reproduced with permission from Ref. ^207^ Copyright 2016 American Chemical Society

Recently, different kinds of nanomaterials have been investigated that are known for better NIR radiation absorption. The first and foremost materials that are widely used are gold and silver nanoparticles due to their suitable behavior.^193^ Surface plasmon resonance, an event where collective electron excitation occurs in these metal nanostructures, has been intensely explored to improve the optical properties of materials, such as the absorption of NIR radiation, scattering, and transition. Notwithstanding the immense efforts of researchers to improve metal nanomaterials for PTT, their aggregation in the human body is of great risk that restricts their large‐scale application.^194^ For such an instance, many researchers have focused their studies on the employment of various structures, such as carbon nanomaterials. Graphene and CNTs possess spectacular optical properties and have turnout as potent remedial agents for PTT.^195^ Nonetheless, their 1‐ and 2‐D structures are known to cause intrinsic toxicity to body tissues and cells.^196^ Recently, the development of organic nanomaterials as PTT agents has attracted significant attention from many researchers. Nanostructures based on polymers have been extensively utilized in nanomedicine for different applications, such as tissue engineering,^197^ drug delivery,^198^ biosensors,^199^ antibacterial materials,^200^ and biointerfaces.^201^ Different polymer nanomaterials acquire prime requirements needed for PTT agents, that is, appropriate shape and size, better dispersion in aqueous medium, quick light to heat conversion after NIR absorption, and biocompatibility in human tissues.^202^ Polymer‐based nanomaterials are mostly used in PTT, not because of their biodegradablility, but they are more advantageous than inorganic nanomaterials due to their facile synthesis and can be used in different forms of treatment.^203^ Fabrication of biomaterials has been performed by using advanced techniques, such as electrospinning and 3D bioprinting for biomedical applications.^204^ Polymer‐based tuned nanomaterials are activated (heat liberated) upon light irradiation, which acts as a moderator to produce transformations in polymer structures, such as contraction/expansion, mobility, crystallinity, or material damage, and thus provide functionalities, such as on‐off switching for the delivery of drugs and genes or other imaging applications.^205^ Wang et al. developed photothermal Pdots based on diketopyrrolopyrrole bridged with conjugated polymers with different thiophene units, which are strong NIR absorbers and display a higher photothermal conversion efficiency of 65%, which is sufficient for the excision of tumor cells both in vitro and in vivo.^206^ Li et al. synthesized dispersible poly(ethylene glycol)‐coated copper nanowires as novel PTT agents. PEGylation was carried out by the addition of 200 mg PEG in 15 mg of a copper nanowire dispersion in 15 ml of THF at 50°C under stirring for 4 h. These PEGylated nanowires possess broader malleability than free nanowires and are interlaced within cancer cells. The heat produced to the cancer cells upon irradiation causes cancer ablation efficiently (Figure 9A). The interaction of PEGylated CuNWs with cancer cells has also been revealed from SEM analysis (Figure 9B) after the incubation of CT26 cells with PEGylated CuNWs for 1 h with and without exposure to NIR laser irradiation for 6 min. Numerous PEGylated CuNWs were observed to enlarge round the cells, indicating the flexibility of PEGylated CuNWs to remain in close contact at the cellular surface. Membrane and cell destruction was evident, which accounted for the transmission of heat directly to the cells, leading to denaturation of protein. Intratumoral administration of PEGylated CuNWs to mice‐bearing colon tumors and immediate exposure to NIR radiation for 6 min increased the temperature to > 50°C, leading to tumor growth inhibition (Figure 9C). To confirm the induced necrosis, immunostaining of the specimen with the necrosis marker HMGB1 was performed (Figure 9D), where the cells treated with PEGylated CuNWs in combination with NIR irradiation released HMGB1 in good quantities compared with the other groups.^207^ Zhang et al. prepared chitosan carboxymethyl chitosan (CMCS)‐coated GN‐based therapeutic agents for photothermal abscission of HepG2 hepatocellular carcinoma cells and HDF human dermal fibroblast cells. First, a GN suspension was synthesized followed by functionalization using CMCS. The highest efficiency of photothermal ablation was observed in in vitro tests by incubating HepG2 and HDF cell lines with CMCS GNs for 2 h and exposing them to an NIR laser (5 W cm^−2^) for 2 min. The results showed complete killing of HepG2 cells and approximately 90% killing of HDF cells upon laser treatment.^208^

### 3.5 Photodynamic therapy

PDT is a clinically proven therapeutic procedure with minimal invasiveness that has emerged for cancer therapy.^209^ PDT induces cell killing in two steps. The first step involves the absorption of visible light by the chemotherapeutic agent, which is particularly accumulated in tumor tissues. Subsequently, light of appropriate wavelength (in which all biological tissues are transparent) is irradiated penetrating deeper inside. In the presence of light, electron transfer takes place to molecular oxygen to form singlet oxygen and other ROS. These generated ROS species activate certain mechanisms that make PDT the most effective technique, such as setting off vascular function resulting in massive cell killing^210^ due to generated oxidative stress.^211^ PDT is an embranchment of physical and life sciences and has been successfully applied in oncology due to combined efforts from chemistry and pharmacology. Compared with conventional chemotherapy and radiotherapy, the PDT‐based treatment procedure for cancer significantly minimizes the toxic effects and enhances the efficacy, as only the targeted areas under illumination are being affected/treated.^212^ Bharathiraja et al. fabricated polypyrrole nanoparticles by incorporating bovine serum albumin–phycocyanin complexes, which were stable under physiological conditions. These formulated nanoparticles did not possess any cytotoxic effects toward cancer cells, but effective killing of MDA‐MB‐231 cells was observed upon laser irradiation. The mechanism involved is the generation of ROS by phycocyanin upon illumination, which completely destroys the cells by converting the optical energy into heat energy.^213^ Hydrophobic PS drugs are effectively encapsulated in nanoparticles due to hydrophobic interactions. Biodegradable polymers, such as PLGA, have been widely used to house PS. PLGA nanoparticles have been used for the topical delivery of ALA. These ALA‐loaded nanoparticles are efficaciously internalized in squamous carcinoma cells and moderate the photocytotoxic effects more efficiently than free ALA at a similar dose.^214^

Tsai et al. reported the sustained release of photoporphyrin IX (PPIX) from graft copolymer (poly(N‐vinylcaprolactam)‐g‐poly(D,L‐lactide) [P(VCL)‐g‐PLA] and poly(N‐vinyl caprolactam‐co‐N‐vinyl imidazole)‐g‐poly(D,L‐lactide) [P(VCL‐co‐VIM)‐g‐PLA]‐based micelles for PDT. PPIX was encapsulated in these copolymer micelles for in vitro and in vivo studies. After laser illumination, PPIX was found in the cytoplasm and nucleus of A549 lung cancer cells, showing a phototoxic effect, forming singlet oxygen causing damage. In vivo studies demonstrate the presence of PPIX release from graft copolymer micelles in the blood stream for a longer time and thereby enhance tumor targeting ability by generating ROS that suppress tumor growth.^215^ Singh et al. reported single‐component star‐shaped polymeric nanoparticles with PEG ends containing biotin (targeting unit) and a coumarin fluorophore for specific site and image‐guided treatment. The anticancer drug chlorambucil is also connected to four arms of PEG to attain the synergistic effect of chemotherapy and PDT. Upon irradiation with UV/Vis light, coumarin generates singlet oxygen along with the release of chlorambucil (80%), which results in significant killing of HeLa cells, showing greater cell destruction via the combined effect of both chemotherapy and PDT. Single‐component targeted polymeric nanoparticles specifically accumulate in cancerous cells (HeLa) to a greater extent than in noncancerous L929 cells.^216^

Chenand coworkers reported H_2_O_2_ activable and oxygen evolving PDT nanoparticles (HAOP NPs) for regulating singlet oxygen release in cancerous cells, attaining sufficient O_2_ in PDT (Figure S2A). Selective uptake of HAOP NP by avb3 integrin rich cancer cells allowed the intracellular penetration of H_2_O_2_ inside the core and was catalyzed by catalse‐generating oxygen leading to cell rupture and release of PS. The H_2_O_2_‐triggered release of methylene blue from HAOP NPs (with/without catalase) is presented in Figure S2B, where 80% release of MB was observed. Moreover, MB release in the absence of catalase was slower, resulting in meagre release in a similar time frame, and comparable results are presented from HAOP NPs with catalase in the absence of H_2_O_2_, revealing that higher release is triggered by H_2_O_2_. The morphology of HAOP NPs with and without catalase during MB release was studied through SEM and is presented in the inset of Figure S2B. The structure of the HAOP NPs with catalase in the absence of H_2_O_2_ and HAOP NPs without catalase in the presence of H_2_O_2_ was preserved during the whole course, while HAOP NPs with catalase ruptured the PLGA shell in the presence of H_2_O_2,_ which is attributed to the release of gaseous oxygen. Cell killing by HAOP NP‐mediated PDT was assessed in cellular studies with the dual fluorescence of annexin V‐FITC/propidium iodide (PI). U87‐MG cells are treated with NPs and subsequently irradiated with a 635 nm laser at a dose of 30 J.cm^−2^, which exhibits intense fluorescence with apoptotic characteristics after irradiation and staining with annexin V‐FITC/PI, indicating apparent cell death compared to cells treated without HAOP NPs and laser irradiation. No fluorescence of annexin VFITC/PI was observed in the cells treated with HAOP NPs (without catalase), and irradiation indicated that catalase was essential for H_2_O_2_‐activated phototoxicity (Figure S2C).^217^ GNs have been used extensively for the delivery of PS drugs at the desired site for either active or passive targeting.^218^ Multifunctional photosensitizer Ce6‐loaded gold vesicles (GVs) were used with trimodality fluorescence, thermal and photoacoustic imaging‐guided synergistic PTT/PDT for the treatment of cancer. These GVs in the NIR region simultaneously exhibit strong fluorescence and NIR radiation excitation of both GVs and Ce6, generating heat and singlet oxygen for synergistic PTT and PDT to significantly kill cancerous cells. In vivo studies using feasible GV‐Ce6 for trimodality fluorescence/thermal/PA imaging synergistic PTT/PDT led to clear visibility of tumor tissues. Thus, synergistic PTT/PDT treatment improves the efficacy in the presence of NIR irradiation.^219^

Radiotherapy remains an important and effective mode of curative treatment for cancer patients, where localized malignant tumors are exposed to radiation, which is usually high‐energy X‐rays. The success of radiation therapy depends on the total radiation dose irradiated at the tumor site. The disadvantage of radiation therapy is that the tolerance of normal tissues near the tumor area limits the dose delivery efficiency for tumorocidal results. Robert R Wilson suggested that accelerated protons can effectively be used for localized cancer therapy based on their favorable penetration depth and dose distribution. The first clinical use of protons for suppressing pituitary hormones in metastatic breast cancer through proton‐based therapy was performed in 1950 at Havrad.^220^ Cancer therapy using heavy ions came into existence in the 1970s in Berkeley by Cornelius, suggesting that particles that are heavier than protons can be more effective. Heavy ions can reduce lateral scattering compared to protons, which enhances the dose distribution at the targeted site.^221^ Another important benefit drawn from their radiobiological effects on cancer tissues is that the effectiveness of charged particles increases with particle ionization density or linear energy transfer.^222^

Recently, hadron therapy has emerged and has become one of the most important medical procedures for treating solid tumors compared to conventional radiotherapy.^222^ Numerous materials have been taken into consideration for this treatment procedure, especially ion species, and at present, mostly protons and carbon ions have been proven to be clinically efficient owing to their satisfactory results in cancer therapy. The reason behind the success of hadron therapy against conventional radiotherapy depends on the fact that energy release from the charged particles is within a few millimeters range, which is close to the penetration range (often termed the Bragg peak area). Therefore, such significant characteristics permit targeted damage to tumor cells and prevent healthy cell damage around the tumor area.^223^ Another facility for cancer treatment has been explored to enhance the efficiency of such procedures. For example, boron neutron capture therapy (BNCT) involves the interaction of thermal neutrons and ^10^B nuclei and is a well‐known process for cancer treatment.^224^ In this technique, injection of ^10^B solution is given into the human body, which is immediately absorbed by tissues surrounding the tumor site, generating energetic alpha‐particles. These induced alpha‐particles, due to their short propagation length and high stopping power, preferentially accumulate in tumor tissue, thereby enhancing the interaction with the tumor cells and subsequently damaging them. In contrast to classical hadron therapy, the delivery of the dose by the charged particle is according to the Bragg‐peak curve. In BNCT, a neutron beam is slowly attenuated in the human body, which results in damage to normal healthy tissues that fall in the neutron beam injection path and reduces neutron doses before the interaction of neutrons with boron in tumor cells.^225^

### 3.6 Chemodynamic therapy

Chemodynamic therapy (CDT), first proposed by Bu, Shi, and coworkers in 2016, is a novel cancer therapy that involves treatment using Fenton or Fenton‐like reactions producing •OH radicals at the tumor site. Later, with the quick progress of Fenton and Fenton‐like nanomaterials, CDT has gained significant attention due to its unique benefits, including its high selectivity for tumors with minimal side effects. The CDT process does not require external field stimulation; it can temper the hypoxic and immunosuppressive tumor microenvironment. CDT is economic as well. Many other metal elements, including copper, manganese, cobalt, titanium, vanadium, palladium, silver, molybdenum, ruthenium, tungsten, cerium, and zinc, have also been used to produce Fenton‐like reaction‐mediated CDT strategies, other than Fe‐mediated CDT reactions. Moreover, CDT has been combined with other therapies, such as chemotherapy, radiotherapy, phototherapy, sonodynamic therapy, and immunotherapy, to achieve enhanced anticancer effects.^226^

Li et al. developed a starvation/chemodynamic therapeutic gel to fight against residual IDH1 (R132H) tumor cells after surgery. In brief, nanoparticles composed of glucose oxidase (GOx) mineralized with manganese‐doped calcium phosphate forming GOx@MnCaP were prepared and then encapsulated into fibrin gel (GOx@MnCaP@fibrin). The fabricated gel was then sprayed at the surgical site, where oxidation of glucose is catalyzed via GOx in residual IDH1 (R132H) cells and produces H_2_O_2_, which is further converted to deadly hydroxyl radicals (•OH) by a Mn^2+^‐mediated Fenton‐like reaction to kill the residual IDH1 (R132H) cells.^227^

### 3.7 Gas therapy

Emerging advances in nanomedicine and nanotechnology have substantially made gas‐based treatment possible for cancer treatment through targeted delivery and controlled release of therapeutic agents. The combination of gas therapy with other treatment methods can sensitize different therapy modes to augment cancer therapy. Basic understanding of the mechanism through which gas enhances other therapeutic modalities enables the design of reasonable strategies for clinical cancer therapy. The design of novel gas‐releasing nanocarriers and the underlying synergistic mechanisms against cancer are essential for their efficacy.^228^ The investigation of gas therapy techniques is a great approach toward green technology for selective cancer therapy. However, there are some challenges, such as uncontrolled or inadequate gas generation and unclear therapeutic mechanisms. Li et al. developed NIR light‐triggered sulfur dioxide (SO2) generation based on a gas therapy approach and demonstrated the in vivo antitumor therapeutic efficacy. To enhance the high loading capacity, SO_2_ prodrug‐loaded rattle‐structured upconversion@silica nanoparticles (RUCSNs) were constructed without any apparent leakage or conversion of NIR light into ultraviolet light for the activation of the prodrug for SO_2_ generation. Furthermore, SO_2_ prodrug‐loaded RUCSNs displayed enhanced cellular uptake, better biocompatibility, intracellular tracking ability, and high NIR light‐triggered cytotoxicity. In addition, the cytotoxicity of SO_2_‐induced cell apoptosis followed by enhanced intracellular ROS generation ultimately leads to damage to nuclear DNA.^229^

### 3.8 Immune therapy

Compared to traditional treatment methods for cancer (including chemotherapy, radiotherapy, and surgery), immunotherapy for cancer has brought about significant improvements in therapy for patients in terms of survival and quality of life. Immunotherapy has now firmly established itself as a novel pillar of cancer care, from the metastatic stage to the adjuvant and neoadjuvant settings in numerous types of cancer.^230^ Programmed cell death protein 1 (PD‐1)/programmed cell death ligand 1 (PD‐L1) blockade immunotherapy has emerged as a promising strategy to treat both solid and hematological malignancies. Li et al. developed a nanoinducer for efficient cancer immunotherapy with high efficiency and cancer‐specific immunogenic cell death. They developed a leukocyte membrane coated with poly(lactic‐co‐glycolic acid) encapsulating glycyrrhetinic acid (GCMNPs) to improve tumor targeting and tumor‐homing capacity and reduce in vivo toxicity. GCMNPs downregulated glutathione‐dependent peroxidases 4 and induced ferroptosis in AML and CRC cells, thereby increasing lipid peroxidation levels. In vivo studies showed that GCMNPs synergized with ferumoxytol and anti‐PD‐L1 and synergistically improved the T‐cell immune response against leukemia and colorectal tumors.^231^

In brief, a new design of vehicles for drug carriers is discussed for sustained release to achieve better disease control, especially for cancer treatment. Different types of nanocarriers are described based on inorganic and organic moieties with various shapes and sizes. Administration of the drug together with the developed nanocarrier emphasizes superior cancer treatment. The efficacy of the nanocarriers and administrative routes have been shown in in vitro and in vivo studies using animal models, and subsequent side effects are deliberated to understand their merits.

## 4 CONCLUSION AND OUTLOOK

Nanotechnology has been developed as a powerful tool as an efficient drug delivery vehicle for cancer treatment. Here, the currently explored nanocarriers for controlled drug delivery are discussed as a developing approach in cancer therapy as well as the necessity for the development of such strategies in terms of targeted therapy. Different organic nanocarriers, such as polymeric systems, liposomes, dendrimers, polymeric micelles, hydrogels, nanoparticles and different inorganic nanocarriers, such as LDHs, CNTs, graphene, GNs, and mesoporous silica‐based polymeric NPs, have been well explored for the delivery of several chemotherapeutics for different cancer treatments. Therefore, administration of different chemotherapeutic drugs in combination or unaided in an appropriate nanocarrier could be considered as an emerging approach for the treatment of cancer in the future. One should take into account not only the physiochemical properties, materials, and loaded cargo but also the cellular and routes of administration for nanocarriers and other barriers present in the administration route, safety and ease of delivery, efficacy in vitro and in vivo along with stability of the particles.

Nanomedicine has become one of the most promising and advanced strategies for cancer treatment. Numerous publications have suggested that nanomedicine therapeutics are highly efficient both in vitro and in vivo for cancer treatment. However, there are only limited nanocarrier‐based medicines that have been successfully used clinically. There are certain challenges in the fabrication of these nanomedicines to be used clinically, such as safety concerns, regulatory issues, physiochemical characterizations of nanomedicines (shape, size, surface distribution, biodegradability, drug loading, surface chemistry, etc.), and manufacturing issues. Moreover, polymer‐based research will definitely continue to flourish, and scientists need to focus on further designing and amending polymers to solve the issues of photobleaching and short blood circulation life in practical applications.

In the future, advances at the interface of engineering and physical sciences will help provide more innovative techniques that will give physicians and patients the diagnostics, information, and therapeutics to eliminate diseases, including cancer. The integration of these disciplines of engineering, physical sciences, and oncology over the past five decades has been a powerful process toward cancer treatment, leading to a medical and technological revolution. Furthermore, integration of these disciplines has the possibility of expediting cancer diagnosis at a very early stage, which will save on expensive later‐stage and last‐minute treatments of metastatic cancer. The efficacies of any treatment can further be enhanced by using techniques that include normal delivery carriers for immunotherapy and drugs that prepare our own body to fight against cancer. Devices/implants fabricated by engineers can be directly inserted/implanted into tumors through minimally invasive surgery, enhancing the efficacy of chemotherapeutics in vivo and thereby making it economically affordable.

## CONFLICT OF INTEREST

The authors declare no conflict of interest. PM is a member of the Editorial Board for *MedComm*. The paper was handled by another Editor and has undergone a rigorous peer‐review process. PM was not involved in the journal's review of/or decisions related to this manuscript.

## AUTHOR CONTRIBUTIONS

AS prepared the draft manuscript and design of figure assembly, and PM conceptualized the review and prepared the final document.

## ETHICS STATEMENT

Not applicable.

## Supporting information

Supporting information

## Data Availability

Not applicable.
