# Supplementary material for: Nanomedicine and versatile therapies for cancer treatment
Source: MedComm (2020). 2022 Aug 18;3(3):e163. doi: 10.1002/mco2.163 (PMC9386439; doi:10.1002/mco2.163)
Supplement: Supplementary file 1 — Supporting information [file MCO2-3-e163-s001.docx]

**Supporting information**

**Nanomedicine and Versatile Therapies for Cancer Treatment**

Aparna Shukla and Pralay Maiti*

School of Materials Science and Technology, Indian Institute of Technology (Banaras Hindu University), Varanasi 221 005, India.

*Correspondences should be made to Pralay Maiti (pmaiti.mst@itbhu.ac.in)

| **S. No** | **Polymeric Structure** | **Applications** | **Reference** |
| --- | --- | --- | --- |
| 1. | **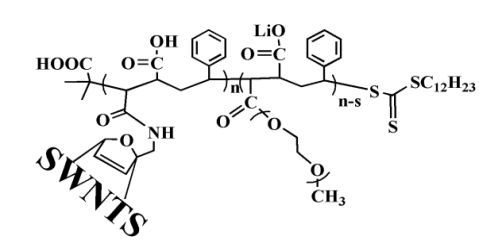**  **PEG-PSMF/SWCNTs** | Faster drug release under acidic tumor micronenvironment and higher antitumor effect towards HeLa cells. | ^1^ |
| 2. |   **HA-g-SS-PZLL** | HA-g-SS-PZLL theranostic nanoparticles shown higher cytotoxicity against HepG2 cells. | ^2^ |
| 3. | **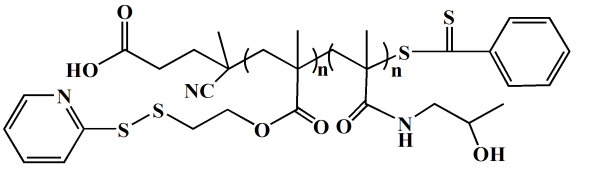**  **Poly(PDSM)-b-poly(HPMA) Block Copolymer** | Faster release of DOX in the presence of a disulfide reducing agent at pH 5.0. | ^3^ |
| 4. | **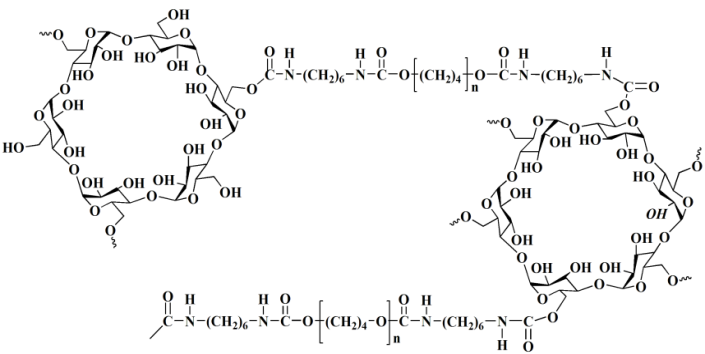**  **Polyurethane graft Cyclodextrin copolym** | CD-g-PU showed controlled drug release and drug loaded polymeric patch exhibited melanoma suppression. | ^4^ |
| 5. | **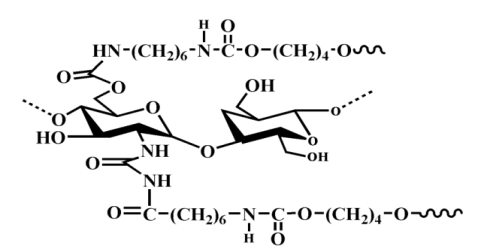**  **PU-g-Chitosan** | PU-g-Chitosan displayed controlled drug release. | ^5^ |
| 6. | **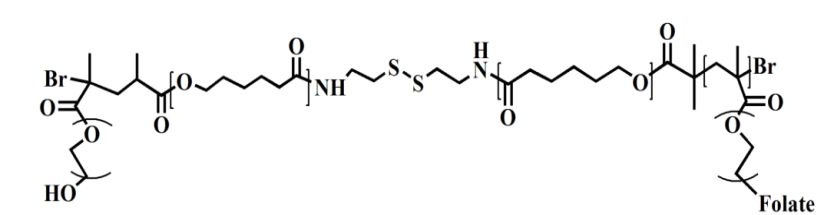**  **Folate conjugated pPEGMA-PCL-s-s-PCL-pPEGMA triblock copolymer** | Redox responsive biocompatible polymer showed enhanced cellular uptake and apoptosisin breast cancer cell line. | ^6^ |
| 7. | 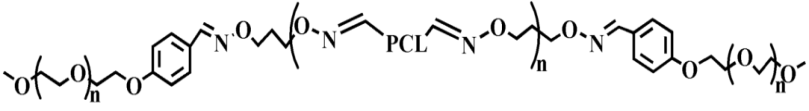  **(PEG-OPCL-PEG) triblock copolymer** | DOX-loaded PEG-OPCL-PEG micelles showed good cell internalization and potent anticancer efficacy. | ^7^ |
| 8. | **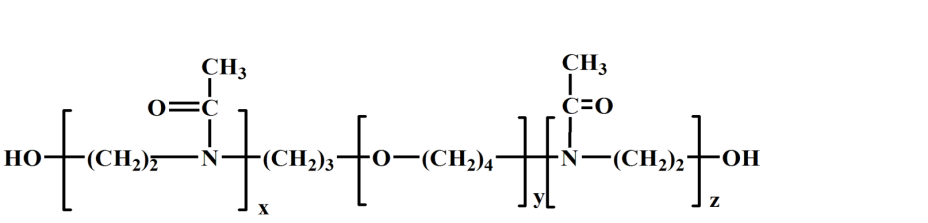**  **pMeOx-b-pTHF-b-pMeOx amphiphilic triblock copolymers** | Demonstratedcell penetration enhancement of a hydrophobic drug by a copolymer of this series was achieved through fluorescence microscopy | ^8^ |
| 9. | **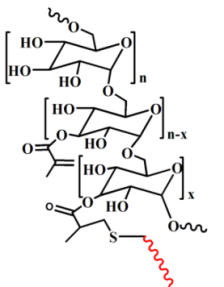**  **Dex−R5H5** | Greater gene expression and much higher cell viability (93%) than PEI/DNA (66%.) | ^9^ |
| 10. | 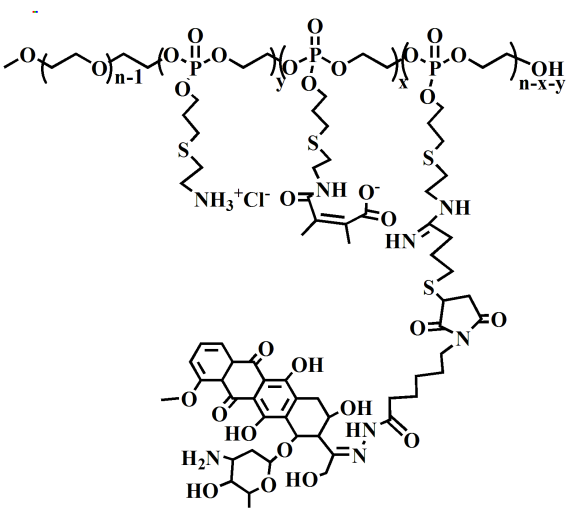  **Polymer-Doxorubicin (DOX) Conjugate (PPC-Hyd-DOX-DA)** | This dual pH sensitive polymer drug conjugate showed enhanced cytotoxicity against cancer stem cells. | ^10^ |
| 11. | **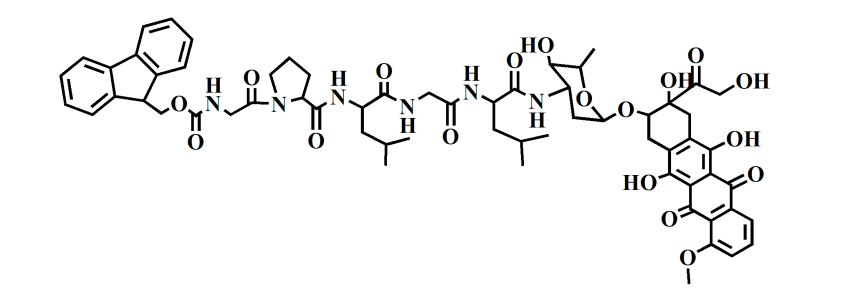**  **Fmoc-GPLGL-DOX(MMP9-DOX)** | The sequential delivery of MMP9-DOX-NPs exhibits enhanced antitumor efficacy against orthotopic 4T1 mammary adenocarcinoma mouse model with reduced systemic toxicity. | ^11^ |
| 12. | ****  **PCL16-b-Kn** | Amphiphilic mimicking antimicrobial peptide copolymers PCL16-b-Kn showed efficient intracellular delivery of DOX. | ^12^ |
| 13. | ****  **P(CM-r-HEMA)** | Significant reduction in IC_50_ was noticed for DOX-loaded Cross linked Vesicless in comparison to free DOX toward MG63 cancer cell lines. | ^13^ |
| 14. | ****  **Dextran-SS-PDP-Biotin** | Biotin-conjugated multistimuli-responsive polysaccharide nanocarriers mediated endocytosis in cancer cells and delivered anticancer drugs to intracellular compartments. | ^14^ |
| 15. | ****  **Degradable Diblock HPMA Copolymer−Drug Conjugates** | Degradable diblock copolymer−gemcitabine (GEM) and −paclitaxel (PTX) conjugates showed the best tumor growth inhibition. | ^15^ |

**Table ST1**: Different polymeric architectures graft, block and polymer drug conjugates and their applications.

**Figure S1: A)** TEM images of C-Dots@MSPs; **B)** Amount of DOX released with time from the nanocomposites; **C)** In-vivo photoluminescence image of nude mice after subcutaneous injection with C-Dots@MSPs; **D)** Fluorescence images of HeLa cells after incubation with C-Dots@MSPs suspension. Color scheme: green, C-Dots@MSPs (a); red, LysoTracker Red (b); yellow, merged green and red (c);Reproduced with permission from ref ^16^. Copyright 2013 American Chemical Society; **E)** Cumulative drug release profile of DOX-loaded CS/PNIPAAm@CNT nanoparticles at different temperatures or pH value; **F)** in vitro cytotoxicity of free DOX, CS/PNIPAAm@CNT nanoparticles, DOX-loaded CS/PNIPAAm@CNT nanoparticles, and DOX-loaded CS/PNIPAAm@CNT nanoparticles with NIR laser irradiation at pH 7.4; **G)** Confocal microscopy images of HeLa cells after 24 h of incubation with free DOX, DOX-loaded NPs, DOX-loaded NPs with NIR irradiation; Reproduced from ref ^17^. Copyright 2015 Elsevier; **H)** Schematic illustration of the synthesis of the multifunctional GO; **I)** FTIR spectra of GO, GO-CMC and GO-CMC-FI- HA; **J)** Release profile of DOX from GO-CMC-FI-HA/DOX under different pH. Reproduced with permission from ref. ^18^. Copyright 2016 Elsevier.

**Figure S2: A)** Schematic illustration of mechanism of H_2_O_2_-controllable release of photosensitizer and O_2_ to implement PDT; **B)** In vitro release profiles of MB from HAOP NPs (with/ without catalase) in the presence or absence of 100 μM H_2_O_2_. Insets SEM micrographs show the morphological changes of HAOP NPs incubated with 100 μM H_2_O_2_ for 24 h. Scale bars= 100 nm; **C)** Confocal fluorescence images of Annexin V-FITC/PI stained U87-MG cells. Reproduced with permission from ref ^19^. Copyright 2103 American Chemical Society.

**References**

1. Cao XT, Patil MP, Phan QT, et al. Green and direct functionalization of poly (ethylene glycol) grafted polymers onto single walled carbon nanotubes: Effective nanocarrier for doxorubicin delivery. *J Ind Eng Chem*. 2020;83:173-180.

2. Yang H, Miao Y, Chen L, et al. Redox-responsive nanoparticles from disulfide bond-linked poly-(N-ε-carbobenzyloxy-l-lysine)-grafted hyaluronan copolymers as theranostic nanoparticles for tumor-targeted MRI and chemotherapy. *Int J Biol Macromol*. 2020;148:483-492.

3. Jia Z, Wong L, Davis TP, Bulmus V. One-pot conversion of RAFT-generated multifunctional block copolymers of HPMA to doxorubicin conjugated acid-and reductant-sensitive crosslinked micelles. *Biomacromolecules*. 2008;9(11):3106-3113.

4. Shukla A, Singh AP, Ray B, Aswal V, Kar AG, Maiti P. Efficacy of polyurethane graft on cyclodextrin to control drug release for tumor treatment. *J Colloid Interface Sci*. 2019/01/15/ 2019;534:215-227.

5. Mahanta AK, Mittal V, Singh N, et al. Polyurethane-grafted chitosan as new biomaterials for controlled drug delivery. *Macromolecules*. 2015;48(8):2654-2666.

6. Kumar A, Lale SV, Mahajan S, Choudhary V, Koul V. ROP and ATRP fabricated dual targeted redox sensitive polymersomes based on pPEGMA-PCL-ss-PCL-pPEGMA triblock copolymers for breast cancer therapeutics. *ACS Appl Mater Interfaces*. 2015;7(17):9211-9227.

7. Jin Y, Song L, Su Y, et al. Oxime linkage: a robust tool for the design of pH-sensitive polymeric drug carriers. *Biomacromolecules*. 2011;12(10):3460-3468.

8. Rasolonjatovo B, Gomez J-P, Même W, et al. Poly (2-methyl-2-oxazoline)-b-poly (tetrahydrofuran)-b-poly (2-methyl-2-oxazoline) amphiphilic triblock copolymers: synthesis, physicochemical characterizations, and hydrosolubilizing properties. *Biomacromolecules*. 2015;16(3):748-756.

9. Tang Q, Cao B, Lei X, Sun B, Zhang Y, Cheng G. Dextran–peptide hybrid for efficient gene delivery. *Langmuir*. 2014;30(18):5202-5208.

10. Du J-Z, Du X-J, Mao C-Q, Wang J. Tailor-Made Dual pH-Sensitive Polymer–Doxorubicin Nanoparticles for Efficient Anticancer Drug Delivery. *J Am Chem So*. 2011/11/09 2011;133(44):17560-17563. doi:10.1021/ja207150n

11. Jiang J, Shen N, Ci T, et al. Combretastatin A4 Nanodrug‐Induced MMP9 Amplification Boosts Tumor‐Selective Release of Doxorubicin Prodrug. *Adv Mat*. 2019;31(44):1904278.

12. Qian Y, Zhou X, He J, Zhou C. Polycaprolactone-based mimetic antimicrobial peptide copolymers vesicles as an effective drug-carrier for cancer therapy. *Polymers*. 2019;11(11):1783.

13. Samanta P, Kapat K, Maiti S, Biswas G, Dhara S, Dhara D. pH-labile and photochemically cross-linkable polymer vesicles from coumarin based random copolymer for cancer therapy. *J Colloid Interface Sci*. 2019;555:132-144.

14. Deshpande NU, Jayakannan M. Biotin-tagged polysaccharide vesicular nanocarriers for receptor-mediated anticancer drug delivery in cancer cells. *Biomacromolecules*. 2018;19(8):3572-3585.

15. Yang J, Zhang R, Pan H, et al. Backbone degradable N-(2-hydroxypropyl) methacrylamide copolymer conjugates with gemcitabine and paclitaxel: impact of molecular weight on activity toward human ovarian carcinoma xenografts. *Mol Pharm*. 2017;14(5):1384-1394.

16. Zhou L, Li Z, Liu Z, Ren J, Qu X. Luminescent Carbon Dot-Gated Nanovehicles for pH-Triggered Intracellular Controlled Release and Imaging. *Langmuir*. 2013/05/28 2013;29(21):6396-6403. doi:10.1021/la400479n

17. Qin Y, Chen J, Bi Y, et al. Near-infrared light remote-controlled intracellular anti-cancer drug delivery using thermo/pH sensitive nanovehicle. *Acta Biomater*. 2015;17:201-209.

18. Yang H, Bremner DH, Tao L, Li H, Hu J, Zhu L. Carboxymethyl chitosan-mediated synthesis of hyaluronic acid-targeted graphene oxide for cancer drug delivery. *Carbohydr Polym*. 2016;135:72-78.

19. Chen H, Tian J, He W, Guo Z. H2O2-activatable and O2-evolving nanoparticles for highly efficient and selective photodynamic therapy against hypoxic tumor cells. *Journal of the American chemical society*. 2015;137(4):1539-1547.
